# Thyroid Hormones and Cancer: A Comprehensive Review of Preclinical and Clinical Studies

**DOI:** 10.3389/fendo.2019.00059

**Published:** 2019-02-13

**Authors:** Eilon Krashin, Agnieszka Piekiełko-Witkowska, Martin Ellis, Osnat Ashur-Fabian

**Affiliations:** ^1^Translational Hemato-Oncology Laboratory, Meir Medical Center, Kfar-Saba, Israel; ^2^Department of Human Molecular Genetics and Biochemistry, Sackler Faculty of Medicine, Tel Aviv University, Tel Aviv, Israel; ^3^Department of Biochemistry and Molecular Biology, Centre of Postgraduate Medical Education, Warsaw, Poland; ^4^Meir Medical Center, Hematology Institute and Blood Bank, Kfar-Saba, Israel; ^5^Sackler School of Medicine, Tel Aviv University, Tel Aviv, Israel

**Keywords:** cancer, thyroid hormone, triiodothyronine, thyroxine, αvβ3 integrin

## Abstract

Thyroid hormones take major part in normal growth, development and metabolism. Over a century of research has supported a relationship between thyroid hormones and the pathophysiology of various cancer types. *In vitro* studies as well as research in animal models demonstrated an effect of the thyroid hormones T3 and T4 on cancer proliferation, apoptosis, invasiveness and angiogenesis. Thyroid hormones mediate their effects on the cancer cell through several non-genomic pathways including activation of the plasma membrane receptor integrin αvβ3. Furthermore, cancer development and progression are affected by dysregulation of local bioavailability of thyroid hormones. Case-control and population-based studies provide conflicting results regarding the association between thyroid hormones and cancer. However, a large body of evidence suggests that subclinical and clinical hyperthyroidism increase the risk of several solid malignancies while hypothyroidism may reduce aggressiveness or delay the onset of cancer. Additional support is provided from studies in which dysregulation of the thyroid hormone axis secondary to cancer treatment or thyroid hormone supplementation was shown to affect cancer outcomes. Recent preclinical and clinical studies in various cancer types have further shown promising outcomes following chemical reduction of thyroid hormones or inhibition or their binding to the integrin receptor. This review provides a comprehensive overview of the preclinical and clinical research conducted so far.

## Introduction

Thyroid hormones (TH) are key regulators of essential cellular processes including proliferation, differentiation, apoptosis, and metabolism. Hypothalamic thyrotropin-releasing hormone (TRH), activates the pituitary gland to synthesize and secret thyroid stimulating hormone (TSH), which in turn acts at the thyroid gland to stimulate TH synthesis and secretion (1). Tetraiodothyronine (T4), the main hormone synthesized in the thyroid gland, is catalyzed to the Triiodothyronine (T3) by specific iodothyronine deiodinases (2). T3 acts as the principal TH mediating metabolic activity, via formation of complexes between T3 and nuclear thyroid hormone receptors alpha (TRα) and beta (TRβ). This nuclear T3-receptor complex binds to thyroid hormone response elements on specific genes, regulating their transcription (3). Diseases associated with excess of TH (hyperthyroidism) and lack of TH (hypothyroidism) are common and present with distinct clinical symptoms.

More than a century ago the association between thyroid hormones and malignancies was first suggested (4). Later, Hercbergs and Leith (5) hypothesized that TH deficiency may affect cancer outcome. This assumption was supported by numerous clinical studies, demonstrating that hypothyroidism inhibits tumor growth, while hyperthyroidism produces an opposite effect (6). A great deal of research has been conducted in recent decades to determine how thyroid hormones exert their growth-promoting effect (7). These mechanisms are now better understood following the discovery of a non-genomic pathway for TH action. αvβ3 is a plasma membrane integrin which acts as a membrane receptor for TH (3). This receptor was shown to contain two distinct binding sites for the hormones, S1 and S2, each translating unique signaling cascades (8). While the S1 site binds solely physiological levels of T3, leading to PI3K activation, the second site, S2, binds T4, and with a lower affinity, T3, activating the ERK1/2 pathway (9). αvβ3 integrin binding facilitates the hormones proliferative action on cancer cells as well as blood vessel cells (3).

In this review, we will provide a summary of studies which examined the link between thyroid hormones and cancer. We will first present the preclinical research on the effects of TH in various cancer models, both *in vitro* and *in vivo*. We will then outline clinical studies examining various aspects of this association including the effect on cancer risk, behavior and outcome.

## *In vitro* Studies of the Thyroid-Cancer Association

This section summarizes the *in vitro* studies on thyroid hormone-cancer association, presented in Table 1. A comprehensive list of the *in vitro* studies, including cancer cell lines and thyroid hormone concentrations, is presented in Supplemental Table 1.

**Table 1:** Preclinical studies on thyroid hormones and cancer.

**Cancer type/model**	**Preclinical studies**	**Thyroid hormones/Inhibitors effect**	**Proposed mechanism**
Breast	*in vitro*	T3 (10–13) and T4 (14) promoted proliferation T3 stimulated cell migration (15). T4 stimulated PDL1 expression (16). Nanotetrac sensitized to apoptosis and increased angiogenesis inhibitors (17) and reduced PDL1 expression (16)	ER mediated (11–13), TR mediated (10), Membrane receptor (14), Integrin αvβ3 (15–17)
		T3 enhanced apoptosis (18). T3 (19–21) and T4 (19, 21) decreased proliferation. T3 enhanced chemosensitization (22, 23)	SMP30 downregulation (18), c-fos upregulation (19), ER expression (20), sensitization of mitochondrial metabolism (22)
	*in vivo*	T3 treatment increased tumor incidence (24). Hyperthyroidism increased tumor incidence (25, 26) and aggressiveness (26). Hypothyroidism decreased tumor incidence (25–31), increased latency (30), slowed tumor growth (30–32), decreased tumor volume (29) and resulted in disease remission (33)	
		Hypothyroidism enhanced invasiveness and metastases (32)	
Prostate	*in vitro*	T3 induced proliferation (34). T4 induced cell migration (35, 36)	Downregulation of BTG2 (34), integrin αvβ3 (36)
		T3 and T4 inhibited proliferation (19)	c-fos upregulation (19)
	*in vivo*	Hypothyroidism reduced growth rate (37, 38). T3 reduced tumor growth (35)	
Lung	*in vitro*	T4 and T3 induced proliferation (39, 40). T4 induced HIF-1α expression (41)	Integrin αvβ3 (39–41)
	*in vivo*	T4 increased tumor growth (42, 43), angiogenesis (43) and metastases (42). Hypothyroidism slowed tumor growth (38). Tetrac suppressed tumor growth (40)	
Ovary	*in vitro*	T3 (44, 45) and T4 (44–46) induced proliferation. T4 induced HIF-1α expression (41). T3 and T4 involved in EMT (47) and induced MAPK and PI3K gene expression (48). Tetrac, Triac, and T1AM inhibited proliferation and induced apoptosis (49)	Akt pathway (45), integrin αvβ3 (41, 44, 46–49)
		T3 and T4 inhibited proliferation (21, 45)	
Cervix	*in vitro*	T4 induced MAPK (50–52)	Membrane receptor (50–52)
Glioma/ glioblastoma	*in vitro*	T3 and T4 induced proliferation (9, 53), and inhibited apoptosis (54)	Integrin αvβ3 (9, 53, 54)
		T3 induced re-differentiation and inhibited proliferation (55)	Akt pathway (55)
	*in vivo*	Nanotetrac reduced tumor size and decreased vascularity (56)	Integrin αvβ3(56)
Neuroblastoma	*in vitro*	T3 inhibited ras-induced proliferation (57)	TR mediated (57)
Renal	*in vitro*	T3 stimulated proliferation (58)	TR mediated (58)
	*in vivo*	Tetrac reduced tumor size (59)	Integrin αvβ3 (59)
Gastric	*in vitro*	T3 induces VEGF and HIF1α (60)	Akt pathway (60)
	*in vivo*	Hyperthyroidism increased cancer incidence (61)	
Pancreas	*in vitro*	T3 increased cell proliferation, migration, and invasion (62)	
		T3 (45, 63) and T4 (45) inhibited proliferation	Cyclin-CDK inhibition(63)
	*in vivo*	Tetrac inhibited tumor growth and angiogenesis (64)	Integrin αvβ3 (64)
Colon	*in vitro*	T3 promoted cell growth and differentiation (65). T4 promoted cell proliferation (66). T3 and T4 up regulated MDR-1 protein (67). T4 stimulated PDL1 expression (16)	Integrin αvβ3 (16, 66)
	*in vitro*	T3 reduced cell proliferation and increased differentiation (68)	E-cadherin induction (68)
	*in vivo*	T4 increased cancer incidence (69)	
Hepatocellular	*in vitro*	T3 increased migration and invasion (70, 71), and activated MAPK and Akt pathway (72) T4 promoted HCC cell self-renewal (73)	TR mediated (70, 71, 73), αvβ3 integrin (72)
		T3 inhibited cell proliferation (74–76) and invasion (77)	TR mediated (74–77)
	*in vivo*	Hypothyroidism reduced tumor growth (32, 78), number and size of metastases and prolonged survival (78). Hyperthyroidism increased invasion and lung metastases (71)	
		Hyperthyroidism associated with preneoplastic nodule regression (79). Hypothyroidism enhanced invasiveness and metastases (32)	TRβ1 up-regulation (79)
Adrenocortical	*in vitro*	T3 and T4 Induced proliferation (45)	
		T3 and T4 inhibited proliferation (45)	
Thyroid	*in vitro*	T3 and T4 induced proliferation (80)	Integrin αvβ3 (80)
	*in vivo*	Hypothyroidism reduced tumor growth (81). Tetrac inhibited tumor growth (82, 83)	Integrin αvβ3 (82, 83)
Melanoma	*in vivo*	Hypothyroidism increased tumor latency and survival (84)	
Basal cell carcinoma	*in vitro*	T3 reduced growth and induced apoptosis (85)	PKA induction (85)
	*in vivo*	T3 reduced tumor growth (85)	
Ehrlich tumor	*in vivo*	Hyperthyroidism increased tumor size (86) and ascitic volume (87)	
Sarcoma	*in vivo*	Hyperthyroidism increased tumor size and metastases (88)	
Multiple myeloma	*in vitro*	T3 and T4 induced proliferation and viability (89, 90) and increase cell migration and invasion (91)	Integrin αvβ3 (89–91)
	*in vitro*	Tetrac inhibited proliferation and induced apoptosis (92)	Integrin αvβ3 (92)
Leukemia	*in vitro*	No direct effect of T3 and T4 (93)	
Lymphoma	*in vitro*	T3 and T4 induced proliferation and VEGF expression (94)	Integrin αvβ3 (94)
	*in vivo*	Hyperthyroidism increased tumor growth (95, 96) and reduced survival (96)	
Angiogenesis	*ex vivo* (CAM model)	T3 (97, 98) and T4 (97–100) induced angiogenesis. Tetrac arrested tumor related angiogenesis (40, 59, 82, 83)	Membrane receptor (98), integrin αvβ3 (40, 59, 82, 83, 97, 99, 100)

### Breast Cancer Cell Models

*In vitro*, thyroid hormones were shown to induce proliferation of breast cancer cells (10, 11, 14). These growth promoting effects were comparable to that of estrogen (E2) and the proliferative effects of T3 or T4 were blocked by co-administration of an estrogen receptor (ER) antagonist, suggesting a significant cross-talk between the two hormones (11, 12, 14). T3 is able to activate estrogen response elements-mediated gene expression in cancer cells (11). In addition, T3 induces the mRNA expression of the growth factors TGFα and TGFβ in ER positive breast cancer cells, while the ER modulator tamoxifen reverts this effect (13, 101). T3 treatment of ER positive ductal carcinoma cells leads to an increase in P53 and Rb phosphorylation, while an ER antagonist blocks these effects (12). T4 induces serine phosphorylation of ERα, which leads to DNA binding and transcriptional activation by the receptor (14). TH also demonstrates tumor promoting effects irrespective of ER signaling. In aggressive triple negative breast cancer cells, T3 enhances aerobic glycolysis (Warburg effect), a hallmark of transformed cells (22). Via a rapid signaling pathway mediated by the integrin αvβ3, T3 was also shown to regulate actin remodeling and to stimulate breast cancer cell migration and invasion (15). T4 was recently shown to stimulate PD-L1 gene expression and increase PD-L1 protein through activation of ERK1/2, thereby supporting the activity of this defensive checkpoint against immune destruction in breast cancer cells (16). In ER negative breast cancer cells, the αvβ3 inhibitor tetraiodothyroacetic acid (tetrac) hinders thyroid hormone cellular actions initiated via the membrane receptor. Nanoparticulate tetrac induces apoptosis through downregulation of apoptosis inhibitors such as XIAP and MCL1 and upregulation of apoptosis promotors such as CASP2 and BCL2L14. Nanotetrac also increases the expression of angiogenesis inhibitor THBS1 as well as the expression of CBY1, a catenin activity inhibitor, and attenuates Ras-oncogene family members (17). It also reduces the effect of T4 on PDL1 gene and protein expression (16). This further supports the assumption that the growth promoting effects of thyroid hormones in breast cancer are mainly mediated through the membrane receptor αvβ3.

### Prostate Cancer Cell Models

Thyroid hormones have shown disparate effects in prostate cancer cells, depending on the thyroid hormone involved (T3 or T4) and cell line investigated. The proliferation of androgen-dependent, but not androgen-independent prostate cancer cells was enhanced by T3. In androgen-dependent cell lines T3 downregulated the expression of the anti-proliferative protein, BTG2 (34). In low invasive prostate cancer cells, but not in highly invasive cancer cells, T4 induced the acquisition of neuroendocrine-like morphology, VEGF secretion and invasive capacity (35). In these cells, while T3 itself had no effect, isoproterenol-stimulated neuroendocrine-like morphology and invasiveness were prevented in the presence of T3. In another study, migration was enhanced and detachment-induced apoptosis was inhibited by T4 in prostate cancer cells, while tetrac, the αvβ3 inhibitor, reversed these effects through diminished activity of the MAPK pathway and inhibited expressions of XIAP, MMP2 and VEGF, suggesting involvement of the integrin in these effects (36).

### Lung Cancer Cell Models

T4 at physiologic concentrations and T3 at supraphysiologic concentrations increase abundance of proliferating cell nuclear antigen (PCNA) and ERK1/2 activation, markers of cell proliferation, in small cell and non-small cells lung cancer models (39). Interestingly, thyroid hormones led to phosphorylation of ERα, while an ERα antagonist blocked T4 induced PCNA expression, ERK1/2 activation and ERα phosphorylation. This suggests, as demonstrated in breast cancer cells, that thyroid hormone mitogenic effects mediated via the plasma membrane may involve an ERα dependent pathway. Tetrac, as well as pharmacologic inhibition of the MAPK pathway, blocked lung cancer cell proliferation in response to thyroid hormones (39, 40). Moreover, in human non-small cell lung cancer cells, T4 at physiological concentrations enhanced internalization and nuclear translocation of the integrin αv monomer. αv monomer then binds inside the cell nucleus promoters of central cancer-related genes, such as ERα, cyclooxygenase-2, hypoxia-inducible factor-1α (HIF1α), and thyroid hormone receptor β1 (41).

### Gynecological Cancer Cell Models

In ovarian cancer cells T3 at supra physiological and T4 at physiological concentrations induced cell proliferation, survival and viability and led to αvβ3 mediated ERK up-regulation (44, 46). Genes that constrain cell cycle (p21, p16), promote mitochondrial apoptosis (Nix, PUMA), and tumor suppression (GDF-15, IGFBP-6) were inhibited by TH, while a hypothyroid environment attenuated ovarian cancer growth (44). TH were also shown to be involved in αvβ3 mediated epithelial to mesenchymal (EMT) transition in ovarian cancer cells, inducing mesenchymal markers zeb-1, slug, and vimentin, and inhibiting the epithelial markers, e-cadherin and zo-1 (47). This suggests a possible implication for TH in ovarian cancer metastases. The αvβ3 inhibitor tetrac induced ovarian cell apoptosis as well as upregulation of ATM and PARP-1, proteins that coordinate recognition of DNA damage (49). As demonstrated for lung cancer models, αv monomer internalization and nuclear translocation were induced by T4, activating multiple genes involved in cancer promotion (41). Importantly, and comparable with results from breast cancer, crosstalk between integrin αvβ3 and ERα promoted the proliferation of ovarian cancer cells by TH, mimicking functions of E2. Both T4 and E2 promoted nuclear translocation of the integrin αv monomer as well as the phosphorylation of ERα, while the presence of an antagonist for ERα blocked T4-induced ERK1/2 activation, ERα phosphorylation, PCNA expression and cell proliferation (46).

In cervical cancer cells (HeLa), T4 was demonstrated to rapidly induce phosphorylation and nuclear translocation of MAPK (50) and to potentiate EGF and TGFα-induced MAPK activation (51). These effects could not be mediated through TR, as HeLa cells lack these receptors. These effects were reproduced by T4-agarose and blocked by tetrac, suggesting a membrane receptor involvement (50–52).

### Central Nervous System Tumor Cell Models

In glioma cells T4 caused proliferation and upregulation of PCNA and MAPK. This effect was inhibited by tetrac, suggesting mediation by the αvβ3 integrin (53). In another study in glioma cells, T3 and T4, acting on the αvβ3 integrin, induced proliferation and activation of ERK1/2, while only T3 activated Src kinase and its downstream PI3-kinase signaling cascade. These findings suggested that the integrin contains two iodothyronine receptor domains, activating different pathways (9). Resveratrol-induced-apoptosis was inhibited in glioblastoma cells by T4, through interference with nuclear COX-2 and ERK1/2 interaction. This effect was prevented by tetrac (54). Other studies demonstrated conflicting results. In both astrocytoma and glioblastoma cells, T3 promoted re-differentiation. T3 increased cell proliferation and phospho-Akt levels in astrocytoma cells, yet suppressed cell proliferation in glioblastoma cells, suggesting differing effects related to cancer aggressiveness (55). In neuroblastoma cells, T3 inhibited ras-induced proliferation and blocked induction of cyclin D1 expression by the oncogene (57). T3 strongly antagonized the transcriptional response mediated by the Ras/MAPK signaling pathway in neuroblastoma cells expressing TRs.

### Renal Cancer Cell Models

Renal cancer is associated with multiple aberrances of thyroid hormone signaling pathway. These include mutations and altered expression of thyroid hormone receptors, decreased intratumoral concentrations of T3, as well lowered expression and disturbed alternative splicing of type 1 iodothyronine deiodinase (102–106). In contrast to normal kidney cells, which decrease proliferation in response to T3, divisions of renal cancer cell lines are stimulated by TH (58). These different T3 effects are the result of distinct, cell type specific regulation of genes that control cell cycle progression. In renal cancer cells T3 attenuates expressions of E2F4, p107, and p130, while in healthy kidney cells the expression of p107 is stimulated by T3. p107 and p130 are proteins of the retinoblastoma family which enable binding of E2F4 and E2F5 to form CERC (cyclin E repressor complex). During G1 phase, CERC interacts with and negatively affects the activity of promoter of *CCNE1*, encoding cyclin E1, thus repressing proliferation (107). In consequence of these disparate effects on gene expression, T3 accelerates cell cycle progression in renal cancer cells, triggering progression to S phase. In contrast, in normal kidney cells, cell cycle progression is attenuated by T3 (58). These pro-proliferative T3 effects on renal cancer cells were confirmed by an independent study (108).

### Gastrointestinal Cancers Cell Models

T4 and T3 differently influence gastrointestinal cancer cells. In contrast to renal tumors, gastric cancers accumulate T3, possibly due to overexpressed transthyretin that mediates cellular T3 import (60). The increased intracellular T3 concentration directly contributes to cancer progression, by inducing the expression of HIF1α, which in turn activates the expression of proangiogenic VEGF. Interestingly, these T3 effects are mediated by accumulation of fumarate, one of the key intermediates of TCA cycle, acting as an inhibitor of HIF1α degradation. These T3 effects are mediated by rapid non-genomic mechanisms, involving PI3K signaling (60). T4, acting on αvβ3 receptors, stimulates colon cancer cell proliferation and activation of PCNA, cyclin D1, and c-Myc (66). These pro-cancerous T4 effects can be prevented by tetrac and nanotetrac. Furthermore, tetrac and nanotetrac potentiate antiproliferative activity of cetuximab, an anti-EGFR antibody, suggesting potential beneficiary effects of these drug combinations in colon cancer patients (66). These pro-mitogenic effects of extracellular T4 are in sharp contrast to mechanisms initiated by T3. Dentice et al. showed that treatment of colon cancer cells with T3 induces their differentiation with concomitant reduced proliferation (68). T3 activated tumor-suppressive E-cadherin, triggering plasma-membrane localization of beta-catenin, thus preventing its nuclear mitogenic activity. These protective T3 actions are prevented by activated beta-catenin which stimulates expression of type 3 deiodinase and downregulates type 2 deiodinase, thereby reducing intracellular T3 pool. In colon cancer cells, T4 mediated the activation of MDR1, suggesting that thyroid hormones may promote drug resistance mechanisms (67, 109).

The effects of T3 in pancreatic tumor cells depend on tumor type. Proliferation of some, but not all, cell lines derived from highly aggressive pancreatic adenocarcinoma was suppressed by T3 (63). Mechanistically, T3 changed expressions of cell cycle regulators, leading to downregulation of cyclins D1 and E, and upregulation of cdk inhibitors, p21^cip1^ and p27^kip1^. Furthermore, T3 attenuated the activity of cyclin-CDK complexes, which resulted in reduced pRb phosphorylation and G1 cell cycle arrest. In contrast, the proliferation, migration, and invasion of pancreatic cancer cells was stimulated by T3 *in vitro* (62). These results fit observations of patients in which hypothyroidism treated with TH supplementation correlated with increased risk of tumor progression and poor prognosis (62). Thyroid hormones were shown to potentiate cytotoxic effects of chemotherapeutics in pancreatic cancer cells (63).

Conflicting *in vitro* results exist regarding the effect of thyroid hormones in hepatocellular carcinoma (HCC). Several studies demonstrated that T3, acting on the TR, leads to inhibition of cancer cell growth. In HCC cells, T3 downregulated oncogenes CDK2, cyclin E and phospho-Rb (74) and up regulated the tumor suppressor p21 and endoglin (74, 75). T3 also induced DKK4, which suppresses cell invasion and metastatic potential via reduction of matrix MMP2 (77) and downregulated ELF2, a transcription factor associated with tumor growth and cell proliferation (76). *In vitro* experiment confirmed that TRβ1 silencing enhanced proliferation and migration of human HCC cells (79). Conversely, T3 action on TR may increase HCC aggressiveness. A high frequency of somatic point mutations of TRα and TRβ were identified in human HCC samples (110, 111). T3 was associated with increased HCC invasiveness through up regulation of furin (70) and lipocalin 2 (71) in a TR dependent manner. Lipocalin 2 and TRα were both overexpressed in HCC patient samples and correlated with cancer grade, stage, and survival (71). T4 action on TRα promoted HCC cells self-renewal, increased cancer stem-like cells and drug resistance and upregulated NF-kB (73). Finally, T3 binding to integrin αvβ3 in HCC cells, induced growth-promoting effects via ERK1/2 and Akt phosphorylation (72).

### Hematological Malignancies Cell Models

T4 and T3 stimulate proliferation and viability of multiple myeloma (MM) cells by activating αvβ3 integrin receptor, leading to rapid activation of the MAPK signaling pathway (89, 90). This in turn, results in activation of genes involved in proliferation (PCNA), and reduced expression of genes encoding apoptotic regulators such as apafl, caspase-3, puma, and noxa (90). Remarkably, the integrin-mediated TH actions may contribute to progression of MM by changes in adhesion and remodeling of extracellular matrix. Specifically, T3 and T4 increased adhesion of MM cells to fibronectin and activated expression of MMP-9 via a mechanism involving αvβ3 and MAPK (91). These *in vitro* results are of potential clinical importance, since tetrac inhibited MM cell proliferation and induced apoptosis. Furthermore, tetrac sensitized patient-derived MM cells to bortezomib, providing a potential new therapeutic option (92). Tetrac also blocked TH-mediated induction of MMP-9 (91).

TH affect proliferation of T-cell lymphoma (TCL) cells by simultaneous induction of genomic and non-genomic mechanisms (112, 113). The non-genomic mechanisms involve rapid membrane translocation of PKC ζ isoform and activation of ERK and NF-κB. One of the downstream targets of PKC ζ signaling is inducible nitrix oxide synthase (iNOS), a well-known activator of TLC proliferation. Barreiro Arcos et al. showed that intracellular activity of TH is prerequisite for activation of iNOS expression, along with enhanced expression of TRα (113). Non-genomic TH actions also contributed to survival and progression of TCL. Specifically, binding of TH to αvβ3 receptors, triggered pro-proliferative, and proangiogenic signals including enhanced expression of cyclins, PCNA, and VEGF. This TH-induced secretion of VEGF stimulated proangiogenic activity of endothelial cells, possibly contributing to TCL progression (94). In another study, *in vitro* treatment of lymphoma cells with T3 and T4 activated proliferation, as indicated by increased expressions of PCNA, as well as cyclins D, A2, and B (95).

### *In vitro* Studies in Other Cancer Models

The cancers originating from thyroid gland are influenced by its own secretory products. Specifically, T4 and T3 promote proliferation of follicular and papillary thyroid cancers *in vitro* (80). These effects are largely mediated by non-genomic signaling involving αvβ3 receptor as indicated by tetrac blockade of the TH-induced proliferation. Furthermore, T4 treatment blocked pro-apoptotic signaling induced by external stimuli such as resveratrol (80). Altogether, this data indicates pro-cancerous and anti-apoptotic role of TH in thyroid cancers. An inhibitory role of TH was reported for skin cancer. T3 inhibited proliferation and induced apoptosis in basal cell carcinoma cells (85). Mechanistically, T3 reduced protein stability and transcriptional activity of Gli2, an oncogenic transcription factor, that promotes G1/S cell cycle phase transition.

Moriggi et al. tested TH influence on six cell lines derived from various types of cancer that differed by the profile of mutations in genes involved in PI3K and beta-catenin signaling pathways. Remarkably, for each cancer type, T3 exerted dual effect, either stimulating or attenuating proliferation. Unfortunately, the presented data did not clarify the cause of this differential T3 effects (45). The results of this study underscore the complexity of mechanisms involved in TH-mediated effects in cancer cells.

Taken together, the results of the *in vitro* studies suggest that effects of thyroid hormones in cancer are mediated by complex genomic and non-genomic signal transduction pathways and are highly dependent on cell type and molecular context. These biological pathways were extensively summarized in a recent review by Goemann et al. (114).

The complex and often contradictory T4 and T3 effects observed *in vitro* underline the importance of *in vivo* studies, which can provide valuable information on the net TH effects in a living organism. On the other hand, *in vitro* experiments provide a unique opportunity to reveal mechanistic details of intra- and extracellular processes initiated by T4 and T3 in cancer cells. Inevitably, both types of studies are required to clarify the role of TH in cancer development and progression.

## *In vivo* Studies of the Thyroid-Cancer Association

This section summarizes the *in vivo* studies on the thyroid hormone-cancer association, presented in Table 1. A comprehensive list of *in vivo* studies is presented in Supplemental Table 2.

### Breast Cancer Animal Models

One of the earliest reports analyzing *in vivo* the link between thyroid hormones and breast cancer was published in 1946. Treatment of mice with the thyroid synthesis inhibitor, thiourea, delayed development of spontaneous breast tumors (115). Similar results were achieved when mice were treated with another compound, thiouracil (116). These results were further validated by Vonderhaar et al., who found that thiouracil-induced hypothyroidism delayed development and decreased incidence of spontaneous breast tumors in mice (27). The study suggested that hypothyroidism could contribute to local atrophy of mammary glands, resulting in reduced tumor formation. Contrasting results were obtained on experimental Ehrlich tumors (ET) that arise from mouse mammary adenocarcinoma (86). In that study, hyperthyroidism decreased metabolic activity and proliferation of ET as evidenced by lowers nuclear diameter, mitotic index, and number of nucleolus organizer regions. TH effects were also tested *in vivo* in models of chemically induced breast tumors. Early studies brought inconclusive results, showing that both thyroidectomy and thyroxine supplementation reduced incidence of breast tumors (117). However, a series of later reports clearly demonstrated the protective effect of PTU (propylthiouracil)-induced hypothyroidism. PTU given at a dose that produced severe hypothyroidism in rats, dramatically reduced the incidence of 7,12-dimethylbenz(a)anthracene (DMBA) (28) and N-methyl-N-nitrosourea (MNU) (29) induced breast tumors. A more recent study further demonstrated that PTU-induced hypothyroidism delayed development and reduced incidence of DMBA-induced mammary tumors by activating apoptosis (30). The protective effects of hypothyroidism were also shown in a model of breast tumor xenografts. Treatment with PTU inhibited growth of inoculated mammary adenocarcinomas and improved survival of mice (31). Spectacular effects of PTU treatment were reported by Shoemaker and Dagher who demonstrated complete remission of mammary tumor xenografts in 77% of PTU-treated mice (33). Confounding reports on the influence of thyroidal status in human cancer was partially explained by Martínez-Iglesias et al. (32). Their study revealed that while the growth of breast cancer cells inoculated into hypothyroid hosts was delayed, the tumors were more invasive and metastatic. The tumors grown in hypothyroid animals were more undifferentiated, with reduced expression of epithelial markers (e.g., keratin 8/18, β-catenin) and enhanced expression of mesenchymal markers (vimentin). However, the same study demonstrated that hypothyroidism reduces cancerous proliferation and stimulates necrosis in tumors, resulting in retarded tumor growth (32). In a parallel study, the same group showed that overexpression of thyroid hormone receptor β (TRβ) attenuated growth of breast tumor xenografts in mice, indicating its tumor suppressive activity (118). These studies demonstrated that intracellular and extracellular effects of thyroid hormones can differently contribute to development and progression of breast cancer, affecting both cancer cells and tumor stroma.

### Prostate Cancer Animal Models

Consistently with results described above, PTU-induced hypothyroidism attenuated growth of prostate cancer xenografts in athymic mice (37, 38). The latter study clearly demonstrated that PTU did not affect the proliferation of prostate cancer cells *in vitro*, supporting the conclusion that anticancer effect was the result of hypothyroid state of the animals (38). In contrast to these suppressive effects of hypothyroidism, a more recent study reported that treatment with T3 (2.5 μg/day) inhibited growth of prostate tumors inoculated in nude mice (35).

### Lung Cancer Animal Models

One of the early *in vivo* studies on TH effects on lung cancer was performed using a model of Lewis lung carcinoma (3LL), an undifferentiated squamous cell carcinoma that spontaneously developed in the lung of C57/BL6 mouse (42). Hyperthyroidism induced by T4 administration significantly increased growth of tumors inoculated by subcutaneous injections of 3LL cells in mice. In contrast, hypothyroidism triggered by methimazole treatment attenuated tumor growth and increased survival of the animals. Remarkably, T3 and T4 differently affected progression of the disease. The number of pulmonary metastases was reduced by treatments with T3 or methimazole while it was increased by treatment with T4. The reduction of tumor growth by methimazole was probably a result of its direct inhibitory effect on cancer cells, since *in vitro* experiments revealed that methimazole suppressed growth of 3LL cells (42). However, another study showed that hypothyroidism itself can also suppress growth of lung tumors. Treatment with PTU significantly suppressed growth of lung tumors subcutaneously inoculated in mice. This PTU effect was possibly not the result of suppressive effect on cancer cells since in a parallel experiment PTU did not affect growth of prostate cancer cells *in vitro* (38). Interestingly, it was suggested that TH may also affect cancer progression by influencing immune response. In the abovementioned study both T4 and methimazole suppressed the activity of NK cells, while alveolar macrophages were activated by T4 and T3 (42). This data indicates that T4 and T3 have broad effects on lung cancer development and progression, not only via direct effects on cancer cells but also by influencing tumor environment and elements of the immune system. The direct effects of T4 on lung cancer cells are probably the result of non-genomic actions. Interesting data was provided by a large study involving 100 mice with Lewis lung carcinoma tumors in which interactions between thyroid hormone and nitric oxide signaling were analyzed (43). Treatment of mice with T4 resulted in a remarkable increase of tumor weight compared to euthyroid animals. These effects were associated with increased expression of VEGF, suggestive of enhanced vascularization. Furthermore, intraperitoneal injections of tetrac, an antagonist of T4 binding to integrin αvβ3, significantly reduced tumor growth and VEGF expression. These results suggested that pro-tumorous T4 effects in 3LL cells are mediated by αvβ3 integrin receptor (43). Similar data was obtained in non-small cell lung cancer cells in which pro-proliferative T4 actions were blocked by antibody directed against integrin αvβ3 as well as by tetrac (40). These promising therapeutic effects of tetrac were also confirmed in studies involving other tumors (56, 64, 82).

### *In vivo* Studies of Gastrointestinal Cancers

Several lines of evidence indicate that high T4 levels promote gastrointestinal carcinogenesis *in vivo*. In rats, T4 administration increased incidence of chemically induced tumors of colon and stomach (61, 69). TH effects were comprehensively analyzed in models of liver neoplasia. It was shown that hypothyroidism delays progression of experimental Morris hepatoma tumors implanted in female Buffalo Rats (78). Specifically, hypothyroidism induced within 2 weeks from tumor implantation not only attenuated growth of localized tumors but also decreased the number of lung metastases and prolonged survival of the animals. These results were further supported by later studies demonstrating tumor suppressive role of TRβ1 in the progress of hepatocellular carcinoma (HCC). Using rat model of HCC, Frau et al. (79) showed that expressions of TRα1 and TRβ1, along with downregulated expressions of their targets, are decreased in tumors. Notably, downregulation of TRβ1 expression was associated with high proliferative activity of liver cells. TRβ1 expression was also decreased in human HCC tissue samples. In contrast, induction of hyperthyroidism in rats bearing nodules resulted in increased TRβ1 expression and regression of preneoplastic lesions. These results clearly suggest a tumor suppressive role of TRβ1 in HCC. However, contrasting results were published on the role of TRα1 in HCC (71), showing that TRα1 is overexpressed in human HCC and stimulates migration and invasion *in vitro* and *in vivo*. Under hyperthyroid conditions HCC cells expressing TRα1 induced invasion and metastases formation in mice. These effects were mediated via MET/FAK pathways. These results were further confirmed by analysis of human HCC tissue samples in which high expressions of TRα1 were associated with lower patients' survival. Remarkably, no such correlation was observed for TRβ1. The authors suggested that T3/TR could play a dual, oncogenic or tumor suppressive role, depending on the molecular background and stage of the disease. So far, this hypothesis was not supported by experimental data. Curiously, the same research group published contrasting results on the expression of TRs in human HCC tissue samples. In a study published in 2012 they reported decreased expressions of TRs (including TRα1) in HCC specimens and concluded that TRs play a tumor suppressive role (119). Clearly, the role of T3 and TRs in HCC requires further elucidation in independent studies involving both human tissue samples and *in vivo* experiments in mice.

### *In vivo* Studies of Hematological Malignancies

The earliest *in vivo* studies exploring the relations between TH and leukemia brought inconsistent results. One study reported decreased incidence of spontaneous lymphatic leukemia in mice with T4-induced hyperthyroidism when compared with hypothyroid mice treated with PTU (120). The hypo- and hyperthyroid leukemic animals did not differ in their survival rates. However, interpretation of these results is challenging, mainly due to limited information on methods and criteria used for leukemia diagnosis in the animals. In contrast, Morris et al. showed that PTU-induced hypothyroidism attenuated lymphomatous infiltrations in rats compared with euthyroid animals (121). Furthermore, hypothyroidism prolonged survival of mice and rats with transplanted lymphomas, while T4 treatment of euthyroid animals resulted in the opposite effects. Different results were obtained in a study focused on progression of acute stem-cell leukemia in rats. The animals were rendered hypothyroid by several methods, including thyroidectomy and thyroid gland ablation with radioactive iodine. Apparently, none of these treatments influenced growth of subcutaneously inoculated tumors nor affected survival of the animals (122).

Non-genomic TH effects were reported for T-cell lymphomas (TCL), defined as a group of heterogeneous lymphoproliferative disorders. TH stimulated TCL proliferation and angiogenesis by acting through αvβ3 integrin receptor (94). TH binding to αvβ3 triggered activation of VEGF and NF-κB pathways, resulting in stimulation of angiogenesis and proliferation. This study further showed that selective inhibition of αvβ3 with cilengitide attenuates growth of TCL xenografts in mice. An interesting study providing the link between TH, stress, and cancer was published by Frick et al. (93). They showed that chronic stress led to suppression of TH plasma levels which was associated with attenuation of T-cell proliferation in response to mitogens, suggestive of impaired immune functions. Supplementation with T4 protected T-cells against stress-induced suppression of proliferation. More importantly, treatment with T4 prevented stress-induced growth of lymphoma tumors subcutaneously inoculated in mice. The study also suggested that TH antitumor effects could be mediated by PKC isoforms θ and α. Exposition of the animals to stress diminished activation of these PKC isoforms. In contrast, T4 supplementation counteracted stress-induced attenuation of PKC activation.

Thyroid hormone status can have dual effect on lymphoma growth and metastasis as shown by a study in which hyperthyroidism stimulated local tumor growth while hypothyroidism fostered formation of metastatic lesions in kidneys (95). Interestingly, these dual effects of TH on primary and secondary malignancies seem to be a more generalized mechanism since similar observations were made in the above mentioned mouse model of breast cancer. Lymphoma cells inoculated in hyperthyroid animals grew faster, with enhanced tumoral and peritumoral vasculogenesis, and increased expression of PCNA and caspase 3 (96). These effects were associated with shorter survival of hyperthyroid animals when compared with eu- and hypothyroid mice. Similar to the effects described *in vitro* (95), enhanced expressions of PCNA and cyclins D and E was described in tumors grown in hyperthyroid animals, when compared with eu- and hypothyroid animals. Surprisingly, hyperthyroidism stimulated apoptosis, as demonstrated by activation of caspase 3 and Bax. The enhanced metastasis observed in hypothyroid animals could be the effect of changes in immune responses. Hypothyroidism increased the percentage of CD4^+^CD25^+^FoxP3^+^ Treg cells in tumor draining lymph nodes (TDLN). Tregs suppress activation and proliferation of CD4+ and CD8+ T lymphocytes. Likewise, activated CD8^+^ T cells (CD8^+^CD69^+^ or CD8^+^CD44^hi^) were decreased in TDLN of hypothyroid animals. The presence of immunosuppressive Tregs in hypothyroid TLDN possibly contributed to metastatic progression, since depletion of CD8+ cells resulted in enhanced metastasis in mice (95). In contrast, TH could prevent metastasis by activation of apoptosis. Indeed, tumors grown in hyperthyroid animals showed increased presence of apoptotic cells when compared with eu- and hypothyroid mice. Remarkably, no signs of apoptosis were found in highly proliferative regions of tumors, explaining their intense growth. Altogether these results suggest that hypothyroidism creates an immunosuppressive milieu that allows for immune tolerance toward metastasizing tumor cells. Another indication of immune tolerance is reduced accumulation of NK cells in spleens from hypothyroid animals suggestive of reduced ability to remove tumor cells by NK cells (95).

### *In vivo* Studies in Other Cancer Models

Protective effect of hypothyroidism was also shown for uveal melanoma, one of the most common and highly metastatic intraocular malignancies (84). PTU-induced hypothyroidism significantly improved survival of mice with ocular melanoma, in contrast to hyperthyroid animals that demonstrated significantly shorter survival when compared to euthyroid animals. Remarkably, uveal melanoma cells expressed high levels of αv and β3 subunits of the integrin receptor, thus providing the platform for binding of T4 and activation of pro-proliferative intracellular signaling cascades.

Studies on the effects of TH on sarcoma brought conflicting results with early studies demonstrating that thyroid radioablation did not change growth of fibrosarcomas in mice (123), while in another reporting that hyperthyroidism attenuated growth of sarcoma tumors in mice (124). However, these results were later negatively verified by independent studies which showed that T4-induced hyperthyroidism stimulated growth and metastatic progression of sarcoma xenografts in mice, while tumor growth was attenuated in mice rendered hypothyroid by radioablation of the thyroid gland (88).

Antitumor effects of T3 were shown for basal cell carcinoma (BCC), the most common human cancer. Topically applied T3 significantly reduced tumor growth in mouse model of BCC (85). Intracellular T3 levels are regulated by activity of type 3 deiodinase (D3) which degrades T3. Depletion of D3 in skin of BCC bearing mice significantly reduced the occurrence of tumors, suggesting that antitumor T3 actions are mediated via its intracellular activity and not mediated by αvβ3 integrin receptor (125).

In contrast, plasma membrane-initiated TH signaling is well-documented in a mouse model of follicular thyroid carcinoma (FTC). FTC tumors are spontaneously developed by Thrb^PV/PV^ mice in which both alleles of thyroid hormone receptor β bear PV mutation that initially was identified in a patient with thyroid hormone resistance. TRβ with PV mutation are unable to bind T3 and activate transcription. Treatment of Thrb^PV/PV^ mice with PTU reduced the expression of integrin αv subunit, thus leading to attenuation of TH-plasma membrane signaling, including the cascade involving PI3K, AKT, and β-catenin (81). This in turn resulted in inhibition of cancerous proliferation and reduction of tumor growth.

In conclusion, the *in vivo* studies in animal models indicate that TH have broad effects on cancer development and progression. On one hand, local intracellular changes in TH concentrations contribute to proliferation of cancer cells, stimulating tumor growth. On the other hand, extracellular hypothyroid milieu may support cancer progression by attenuating immune responses. Several lines of evidence strongly indicate that non-genomic T4 actions can trigger cancerous proliferation and that interference with T4-αvβ3 integrin binding can provide efficient therapeutic option for patients.

## Clinical Studies of the Thyroid-Cancer Association

This section summarizes clinical studies on the thyroid hormone-cancer association, presented in Table 2. A detailed list of clinical studies, including study design and number of patients, is presented in Supplemental Table 3.

**Table 2:** Clinical studies on thyroid function and cancer.

**Cancer type**	**Thyroid function/ Treatment**	**Clinical outcome**
Breast	Hyperthyroidism	Increased risk (126–129), higher mortality (130, 131). Higher T3 (132, 133) and T4 (23, 132–134) in cancer patients. T3 associated with cancer risk (135, 136), large tumors (135), lymph node metastases (135), and cancer death (137, 138)
		No effect on cancer risk (139–141)
	Hypothyroidism	Decreased risk (129, 142, 143), longer progression free survival (144), later diagnosis (142), more localized disease (142), less lymph node involvement (142). Lower mortality (145). Decreased risk of triple+ BC with higher TSH (146)
		Increased risk (147)
		No effect on cancer risk (139–141, 148, 149)
	LT4 treatment	Increased risk (147, 150)
		Lower all-cause mortality (151)
		No effect on cancer risk (152)
Prostate	Hyperthyroidism	Increased risk (153, 154), T3 associated with risk of recurrence (155). Higher T3 in cancer patients (156)
	Hypothyroidism	Lower risk (154, 157)
Lung	Hyperthyroidism	Increased risk (126, 153). Higher T4 and lower TSH in cancer patients (158)
	Hypothyroidism	Longer survival (159, 160) and later diagnosis (159)
	LT4 treatment	Increased risk (161)
Ovary	Hyperthyroidism	Increased risk (162), higher mortality (130, 163). Higher T4 in cancer patients (164)
Uterine	Hypothyroidism	Increased mortality (165), Elevated TSH associated with lower survival (166)
Central nervous system	Hyperthyroidism	Increased risk (167)
	T3 treatment	Prolonged survival (168)
	Induced hypothyroidism	Prolonged survival (169, 170)
Renal	Hypothyroidism	Increased survival (171–178), increased remission (173), tumor regression (179), and response to treatment (180)
		Increased risk (167)
Esophageal cancer	Hyperthyroidism	Higher incidence in cancer (181)
Pancreas	Hyperthyroidism	Increased risk (182), increased mortality (165)
	LT4 treatment	Higher perineural invasion (62), T stage (62), nodal spread (62) and poorer prognostic stage (62)
	Induced hypothyroxinemia	Tumor regression (183)
Colorectal	Hyperthyroidism	Increased risk (184)
	Hypothyroidism	Increased risk in untreated hypothyroidism (184). Higher subclinical hypothyroidism in colorectal cancer (185). Increased risk with higher TSH (186)
	LT4 treatment	Decreased risk (184, 187, 188)
	Free T3/free T4 ratio	Higher ratio associated with increased survival (189)
Hepatocellular	Hyperthyroidism	Low TSH associated with smaller tumors (190). T3 and T4 inversely associated with cancer mortality (191)
		Lower survival with elevated T4 (190)
	Hypothyroidism	Increased risk (192). High TSH associated with larger tumors (190). Increased incidence in HCC patients of unknown etiology (193)
	TSH x free T4	Higher value associated with favorable time to tumor progression and overall survival if chemotherapy provided and unfavorable TTTP and OS if sorafenib administered (194)
Thyroid	Hyperthyroidism	Increased risk (167, 195)
Head and neck	Hypothyroidism	Increased survival (196–198)
Melanoma	Hypothyroidism	No difference in survival (199)
Multiple myeloma	Hyperthyroidism	T3 higher and TSH lower in patients (200)
Leukemia	Hyperthyroidism	T3 and T4 higher and TSH lower in patients (201). Improved outcome in Grave's disease (202)
	Hypothyroidism	Improved outcome in Hashimoto thyroiditis (202)
Myelodysplastic syndrome	Hyperthyroidism	T3 and T4 higher and TSH lower in patients (203)
General	Hyperthyroidism	Increased risk (126, 153, 195), Increased cancer death in hyperthyroidism (165), and toxic nodular goiter (204)
		T3 inversely associated with cancer mortality (191)
		No association to cancer mortality (205, 206)
	Hypothyroidism	Lower mortality (145, 207), Longer survival (208), High response to radiation therapy (209)
		Increased risk (167), Increased cancer mortality (137)
	Induced hypothyroxinemia	Prolonged survival (210)

### Effect of Thyroid Status on Cancer Risk

#### Effect on the Overall Risk of Cancer

Hellevik et al. conducted a prospective population based study of 26,691 people without a previously diagnosed thyroid disease (153). Baseline TSH levels were measured and 9 years of follow up of cancer incidence was recorded. Compared to euthyroid reference group, increased cancer risk (HR 1.34) was associated with low TSH levels (<0.5 mU/l), a risk driven by lung cancer (HR 2.34) and prostate cancer (HR 4.99). In another population based cohort study, 17,034 patients with newly diagnosed hyperthyroidism were matched with 34,066 patients without hyperthyroidism. Over a 4 year follow up period, patients with hyperthyroidism were at higher overall risk of cancer (Adjusted HR 1.2, *p* < 0.05) and thyroid cancer (Adjusted HR 6.8, *p* < 0.05), with extended duration of hyperthyroidism associated with greater risk of thyroid cancer (195). The Rotterdam study prospectively included 10,318 patients with baseline measurements for free T4 and TSH, followed for a median of 10.4 years. Higher free T4 levels were associated with higher risk of solid cancers (HR 1.42 per unit increase in free T4), lung cancer (HR 2.33), and breast cancer (HR 1.77), although no association were found for TSH levels (126). Collectively, these prospective studies support a causal association between disorders in thyroid hormones and cancer risk.

#### Effect on the Risk of Breast Cancer

In a population based case control study including 676 breast cancer patients and 680 controls (127), free T4 levels were associated with a high overall risk of breast cancer (OR 1.4 for free T4 above vs. below the median). This increase was later attributed to a higher incidence rate of less aggressive breast cancer subgroups (128). Another prospective cohort study conducted by the same group included 2,185 women followed for an average of 19.3 years for breast cancer incidence (135). An association was demonstrated between T3 and breast cancer risk (HR 1.61 of third quartile compared to first). In another large population-based study conducted by Søgaard et al., women with hyper- and hypothyroidism were followed for up to 7.4 years (129). Hyperthyroidism was related to a slight increase in the risk of breast cancer compared to the general population (SIR 1.11), while the opposite was shown for hypothyroidism (SIR 0.94). In a retrospective cohort study of 437 breast cancer patients, elevated levels of TSH were associated with a lower likelihood of triple positive breast cancer (ER+ PR+ Her2/neu+) compared with ER+ PR+ Her2/neu– breast cancer. However, no association was found with tumor grade or stage (146). Interestingly, Brandt et al. were recently able to identify a SNP (rs2235544) in the gene for deiodinase type 1 (DIO1) which was associated with both free T4 level and breast cancer risk (211). A recently published case control study which included 682 breast cancer patients and 731 controls demonstrated an association between higher serum total T4 and breast cancer in both premenopausal (OR 5.98) and post-menopausal women (OR 2.81), whereas a negative association was demonstrated between total T3 and breast cancer (134). Similarly, Huang et al. demonstrated higher free T4 and lower T3 in patients with newly diagnosed breast cancer compared with patients with benign breast lesions (23). However, these findings may demonstrate the effect of malignancy on the thyroid axis, rather than a true demonstration of risk or causality. Specifically, malignancy may be associated with a reduction of T3, resulting the so-called non-thyroidal illness syndrome (NTIS) (212). The association between NTIS and malignancies is later detailed in this review in a designated section. A meta-analysis from 2012, which included 10 case control studies of hyper- and hypothyroidism and breast cancer, failed to find a putative relationship of either disorder. Notably, a high degree of heterogeneity was demonstrated between the six hypothyroidism studies included (139). Another meta- analysis from 2017 included population based studies assessing thyroid dysfunction and the risk of breast cancer. Analysis of 12 studies, including 24,571 cases, also did not find a statistical correlation between hypothyroidism and breast cancer (*p* = 0.162). Similarly, by analyzing 10 studies, which included 21,889 cases, the authors did not demonstrate a statistically significant higher risk of breast cancer in hyperthyroid patients (140).

#### Effect on the Risk of Prostate Cancer

In a prospective cohort study, sera from 3649 patients were assayed for TSH and free T4 (154). During a 20 year follow up period, 7.8% of males were diagnosed with prostate cancer. Higher TSH was associated with a lower risk of prostate cancer (adjusted HR: 0.7 per 1 mIU/L increase in TSH). Similarly, higher free T4 was associated with increased risk of prostate cancer (adjusted HR: 1.11 per 1 pmol/L increase). In a prospective study of male smokers including 402 prostate cancer patients and 800 controls (157), TSH in the highest quintile was associated with decreased risk of cancer (Q5 vs. Q1–4: OR 0.7). Hypothyroid men (high TSH with normal or low T4) had lower prostate cancer risk compared to euthyroid men (OR 0.48).

#### Effect on the Risk of Gynecologic Cancers

In a population based case-control study, 767 patients with recent diagnosis of epithelial ovarian cancer were compared with 1,367 community controls (162). Based on data retrieved from interviews, hyperthyroidism history was linked with increased cancer risk (OR 1.8). In another study, Kang et al. evaluated the association of self-reported history of thyroid dysfunction with medical records of confirmed endometrial carcinoma (*n* = 1,314) and ovarian cancer (*n* = 1,150) as part of the Nurses' Health Study (NHS). In this case, history of hypothyroidism or hyperthyroidism was not associated with cancer risk (213). Lastly, in a retrospective study Brinton et al. assessed the relationship between hospital and outpatient admission for various conditions and subsequent development of uterine and ovarian cancer. A prior diagnosis of thyroid disease was associated with uterine cancer (RR 1.52). However, no specific type of thyroid disease was more strongly linked to risk than others (214).

#### Effect on the Risk of Gastrointestinal Cancers

In a large population based case control study by Ko et al., 532 patients with pancreatic cancer were matched to 1,701 controls randomly selected from the same population (182). Based on patient self-report, hyperthyroidism history was related with increased cancer risk (OR 2.1).

Unlike pancreatic cancer, data regarding colorectal cancer (CRC) and hepatocellular carcinoma (HCC) produced conflicting results. A single prospective population based study suggested an association between hyperthyroidism and an increased risk of CRC (153), but due to a small cohort, this increase was not statistically significant. In contrast, additional studies demonstrated that hypothyroidism was associated with increased risk of colon cancer. A large case-control study compared 20,990 colorectal cancer (CRC) patients and 82,054 matched controls from a population database, followed for an average of 6.5 years and determined CRC risk in patients with thyroid dysfunction (184). In this study both hyperthyroidism (OR 1.21) and untreated hypothyroidism (OR 1.16) were associated with increased risk of colorectal cancer. Chan et al. conducted a prospective case-control study of 3,836 older men (186). Over a median follow up period of 9 years, 136 men developed colorectal cancer. Following adjustments, higher TSH was related with increased incidence of colorectal cancer (SHR 1.19), an association which was reinforced after eliminating the first year of follow up (SHR 1.23). Free T4 was not associated with cancer incidence in this study.

Similar to CRC, hypothyroidism may also play a role in liver carcinogenesis. Hassan et al. conducted a case-control study including 420 patients with hepatocellular carcinoma and 1,104 healthy controls (192). Hypothyroidism of longer than a decade was associated with significantly higher risk of HCC in women, unrelated to known HCC risk factors (OR 2.9 following regression analysis for risk factors). However, the data on thyroid disorders was based on patient self-report using questionnaires rather than thyroid hormone levels. Reddy et al. (193) demonstrated that in 160 patients with HCC, hypothyroidism was more prevalent in HCC of unknown etiology compared to patients with HCC secondary to HCV or alcoholic liver disease (OR 6.8).

The inverse correlation between thyroid function and cancer risk observed in CRC and HCC may be attributed to the elevation of TSH under hypothyroid state. The overexpression of a functioning thyroid stimulating hormone receptor (TSHR), which was demonstrated in HCC tissues (215), may provide a possible mechanism. TSH elevation in hypothyroidism may lead to HCC progression through direct stimulation of its receptor on cancer cells. However, no documentation for a similar TSHR expression in CRC was reported to date.

#### Effect on the Risk of Hematologic Malignancies

Dalamaga et al. compared 73 patients with primary multiple myeloma to 73 matched controls admitted for non-neoplastic conditions (200). The prevalence of clinical thyroid disease was higher in multiple myeloma patients compared to controls (adjusted OR 4.03 for thyroid disease, 5.68 for autoimmune thyroid disease). The levels of free T3 was higher (3.5 vs. 2.7 pg/ml, *p* = 0.002) and TSH lower (2.2 vs. 3.1 μIU/ml, *p* = 0.001) in myeloma patients compared to controls, albeit within the normal range. The same group conducted a case control study of 101 patients with histologically and cytogenetically confirmed MDS to 101 matched control. MDS patient had significantly higher serum levels of free T3 and free T4 and lower TSH than controls (203). A small case control study compared thyroid hormone levels between 25 patients with acute leukemia and 25 matched controls. Total T3, free T3, total T4, and free T4 were higher in patients than control, within the normal range, while TSH levels were significantly lower (201). However, since TH levels were not determined prior to the development of disease, an assessment of risk could not be established.

### Effect of Thyroid Hormones on Clinical Presentation of Cancer

Cristofallini et al. retrospectively compared 1,136 women with breast cancer with 1,088 controls (142). Prevalence of hypothyroidism was significantly lower in the cancer group compared to the control group (7.0 vs. 14.9%, *p* < 0.001). Hypothyroid breast cancer patients were diagnosed at an older age (58.8 vs. 51.1 years; *p* < 0.001), had higher probability for having a localized disease (95.0 vs. 85.9% clinical T1 or T2 disease, respectively; *p* = 0.025), and were more likely to be lymph node negative (62.8 vs. 54.4%; *p* = 0.15). While these findings suggest that hypothyroidism slows breast cancer progression, the study was limited by its retrospective nature and by the fact that the diagnosis of hypothyroidism was based on information from medical charts rather than hormone values. In the aforementioned study by Tosovic et al. (135), higher T3 level (third quartile compared to first) was associated with large breast tumors (>20 mm) (HR of 3.17) and lymph node metastases (HR 4.53). This association was especially pronounced in post-menopausal women. In a series by Atkins et al. 34 patients with various advanced neoplasms (melanoma, renal cell carcinoma, lymphoma, and colon cancer) had received treatment with interleukin-2 and lymphokine-activated killer (LAK) cells (179). Twenty-one percent of patients had laboratory evidence of hypothyroidism. Patients with hypothyroidism had a higher rate of tumor regression (71 vs. 19%, *p* < 0.02).

Conversely, hypothyroidism was associated with increased aggressiveness of colorectal and liver cancer, comparable with the effect on cancer risk. In a case control study comparing 273 colorectal cancer patients to 819 matched controls, the prevalence of subclinical hypothyroidism was significantly higher in the colorectal neoplasm group (24.5 vs. 15.3%, *p* < 0.01). Compared with euthyroid subjects, hypothyroid patients had higher likelihood of advanced colonic disease (8.3 vs. 4.4%, *p* = 0.028) (185). In another study by Pinter et al., 667 patients diagnosed with non-surgically treated HCC were retrospectively followed for a mean period of 65.5 months (190). Hypothyroid patients (TSH>3.77 μU/ml) had a higher risk for large lesions (>5 cm), while Hyperthyroid patients (TSH<0.44 μU/ml) had a lower risk.

### Effect of Thyroid Status on Cancer Survival

#### Effect on Overall Cancer Survival

Several population based studies have demonstrated increased cancer mortality in hyperthyroidism, with opposite outcomes in hypothyroidism, supporting the assumption of growth promoting effect of thyroid hormones. Brandt et al. used data of 2,152 patients with Grave's disease and toxic nodular goiter, followed for 11 years (204). Both diseases were associated with increased all-cause mortality compared with non-hyperthyroid controls, and increased cancer mortality was demonstrated for toxic nodular goiter (HR 1.36, *p* < 0.05). In a recent population based study by Journy et al., 75,076 female radiologic technologists who completed medical questionnaires were retrospectively followed for a median of 28 years (130). No association was demonstrated between overall cancer mortality and hyper or hypothyroidism. However, risk of breast cancer mortality after 60 years of age was increased in patients with self-reported hyperthyroidism (HR 2.04, *p* < 0.05). Women with hyperthyroidism treated with radioactive iodine had increased risk of ovarian cancer mortality compared with women without thyroid disease (HR 5.32, *p* < 0.05), based on very few cases. Lechner et al. (216) conducted a retrospective cohort study of 538 patients with various solid malignancies (renal cell carcinoma, GIST, HCC, neuroendocrine, primary central nervous system, other carcinoma, sarcoma) treated with tyrosine kinase inhibitors. Thirteen percent of patients developed subclinical hypothyroidism and 27% developed overt hypothyroidism. Patients with hypothyroidism had significantly longer overall survival (median overall survival 1,005 days in subclinical hypothyroidism and 1,643 in overt hypothyroidism compared with 685 days in euthyroid patients, *p* < 0.0001). In Franklyn et al.'s retrospective cohort of hyperthyroid patients treated with radioiodine (217), mortality from cancers of all sites was reduced following treatment (SMR 0.9, *p* = 0.02). In subgroup analysis lower mortality was significant only for cancer of the bronchus and trachea (SMR 0.78, *p* = 0.03) while for cancers of the small bowel and thyroid, small absolute risk increases in mortality were demonstrated. Opposing results were demonstrated in a large population based study, wherein 115,746 patients were followed for 10 years for evaluation of cancer mortality (137). Following adjustment, patients with biochemically proved subclinical hypothyroidism (1.6%) at study inclusion had higher risk of cancer death (RR 1.51, *p* < 0.05) as well as increased risk of bone, skin and breast cancer (RR 2.79, *p* < 0.05). A prospective study by Zhang et al. (191) was conducted on a cohort of 212,456 middle aged Korean subjects who had undergone thyroid function tests. Following a median follow-up of 4.3 years, an inverse association was demonstrated between free T4 and all-cause mortality (HR = 0.77, *P* = 0.01) as well between free T3 and cancer mortality (HR = 0.62, *p* for trend = 0.001). TSH was not associated with mortality endpoints. This discrepancy may be at least partially attributed to the large proportion of gastrointestinal tumors in both studies (>45% of cancer deaths) which, as described above, may propagate in hypothyroid conditions.

#### Effect on Breast Cancer Survival

In 1964, Humphrey and Swerdlow were among the first to demonstrate the effect of thyroid disorders on breast cancer outcomes (218). In their study, the 5-year survival of 14 patients who had undergone a thyroidectomy for non-toxic goiter was significantly longer than nine patients who had undergone thyroidectomy for hyperthyroidism (71 vs. 22%, *p* < 0.05). In another study of 462 cases of breast cancer (131), patients with a history of thyroid disease had significantly lower survival rates at 5 and 10 years compared with controls (*p* < 0.005). In a subgroup analysis, patients with a history of treated hyperthyroidism had significantly shorter 5- and 10-year survival (*p* < 0.01). A population-based prospective cohort study by Tosovic et al. included 2,185 women who had T3 levels measured as part of a preventive health study (138). After a mean follow-up of 24.1 years, 26 women died of breast cancer. T3 levels were correlated with age-adjusted breast cancer related death (HR 2.8, *p* = 0.012), especially in post-menopausal patients (adjusted HR 3.73, *p* = 0.001). Thyroid hormone status may also affect response to breast cancer therapy. A study by Cao et al. included 28 patients with metastatic breast cancer treated with the VEGFR-2 inhibitor famitinib (144). Sixty-four percent of patients had elevated TSH (>4.94 mIU/L) during treatment. Progression free survival (PFS) was longer in these patients compared with patients with normal TSH (107 vs. 53 days, respectively, *p* = 0.002).

#### Effect on Lung Cancer Survival

Several decades ago, Hercbergs and Leith reported a case of a 69 year old male with metastatic non-small cell lung cancer that resolved spontaneously following resuscitation from myxedema coma, dying of unrelated causes 4 years after the myxedema event (5). In a later retrospective case-controls study (159), Hercbergs et al. compared 85 hypothyroid lung cancer patients to 85 matched euthyroid lung cancer patients. Hypothyroid patients were older at diagnosis (median age 73 vs. 64 years, *p* = 0.0006) and survived longer (stages 1–4: 14.5 vs. 11.1 months, *p* = 0.014; stage 4: 11 vs. 5 months, *p* = 0.0018) compared with controls. In a recent study of 51 non-small-cell lung cancer patients treated with pembrolizumab, a PD-1 inhibitor (160), 21% of patients developed hypothyroidism requiring thyroid hormone replacement, with 80% developing positive antithyroid antibodies. Overall survival (OS) with pembrolizumab was significantly longer in subjects who developed thyroid dysfunction (mean OS 40 vs. 14 months. HR 0.29, *p* = 0.04).

#### Effect on Ovarian Cancer Survival

Minlikeeva et al. used collective data from 11 studies, including information on thyroid hormone status for a total of 5,822 patients diagnosed with invasive ovarian cancer (163). Increased risk of mortality was demonstrated for patients with a history of hyperthyroidism in the 5 years preceding cancer diagnosis (HR = 1.94; *p* = 0.01). Hypothyroidism was associated with a mildly decreased mortality risk (HR = 1.16; *p* = 0.01). Duration of hypothyroidism or thyroid medications use were not associated with survival.

#### Effect on Renal Cancer Survival

Much research has been conducted on the possible positive effects of drug-induced hypothyroidism on the outcome of treatment for renal cell carcinoma (RCC). In a small scale study by Weijl et al. (180), patients with metastatic renal cell carcinoma were treated with IL-2 and LAK cells. Forty-seven percent of patients became hypothyroid following treatment. Favorable response to treatment was positively correlated with hypothyroidism (*r* = 0.76, *p* = 0.001).

Over the past decade, several studies have reported on potential favorable outcomes of tyrosine-kinase inhibitors (TKI's)-induced hypothyroidism, specifically by sunitinib. Sunitinib is an oral multitargeted tyrosine kinase inhibitor commonly used in metastatic renal cell carcinoma. Hypothyroidism is a common side effect of this treatment, with up to 85% patients developing abnormality of thyroid function consistent with hypothyroidism, and roughly a third requiring thyroid hormone replacement (219, 220). The mechanism for this effect is not altogether clear, though it may be related to a decreased VEGF binding to normal thyroid cells and/or disruption of thyroid blood flow (219). In a retrospective analysis of metastatic RCC patients who received VEGF receptor tyrosine kinase inhibitors (171), median OS and PFS were significantly longer in patients with a peak TSH >10 mIU/L compared to patients with a peak TSH of ≤ 10 mIU/L (not reached vs. 21.4 months, *p* = 0.005; 47.7 vs. 9.3 months, *p* = 0.009, respectively). In a series of sunitinib-treated clear cell RCC (172), hypothyroid (TSH >4 mIU/L) patients receiving levothyroxine as thyroid-replacement therapy had prolonged PFS compared with other patients (25.3 vs. 9.0 months; *p* = 0.042). A prospective cohort study by Schmidinger et al. included 87 sunitinib or sorafenib treated patients with metastatic RCC (173). Patients who developed subclinical hypothyroidism had a higher rate of remission compared with euthyroid patients (28.3 vs. 3.3%, *p* < 0.001) and longer median duration of survival (not reached vs. 13.9 months, *p* = 0.016). Other studies have similarly demonstrated prolonged PFS in sunitinib induced hypothyroidism (174, 175). One prospective study did not show such a correlation (221). However, this study was based on only 6 months of follow up. In 2015, Nearchou et al. published a meta-analysis evaluating hypothyroidism as a predictive marker for survival in metastatic RCC patients treated with TKI's (176). Based on six studies, PFS in patients with sunitinib-induced hypothyroidism was not significantly different compared with patients without hypothyroidism. However, in three studies which included patients treated with sunitinib or sorafenib, the difference in PFS was statistically significant in favor of patients with acquired hypothyroidism (HR, 0.59; *p* = 0.003). Moreover, an analysis of four studies indicated a statistically significant improvement in OS in patient who developed sunitinib-induced hypothyroidism compared with patients who did not (HR 0.52, *p* = 0.01).

#### Effect on Gastrointestinal Cancer Survival

Similar to the effect on cancer risk, CRC and HCC appear to represent cancer subtypes whose association with thyroid status differs from that of other solid tumors. In a recent study by Schirripa et al. (189), a higher baseline free T3/free T4 ratio was associated with increased survival in patients with metastatic colorectal cancer treated with the multikinase inhibitor regorafenib (*p* = 0.003). In Pinter et al.'s study of non-surgically treated HCC patients (190), increased OS was associated with lower TSH (≤ 1.7 vs. >1.7 μU/ml, median OS 12.3 vs. 7.3 months; *p* = 0.003) and lower free T4 (≤ 1.66 vs. >1.66 ng/dl, median OS, 10.6 vs. 3.3 months; *p* = 0.007). Similarly, in a study by Zhang et al. (191), both free T3 and free T4 were inversely associated with liver cancer mortality (HR per SD change: 0.64 for free T3, 0.52 for free T4). These findings again support a growth promoting effect of hypothyroidism in HCC, which may be related to activation of the TSH receptor. In a recent case control study (194), the product of TSH and free T4 was calculated for 123 patients with advanced HCC treated with sorafenib or chemotherapy. High TSH x free T4 at baseline (>2.48) was associated with favorable time to tumor progression (TTTP) (HR 0.478, *p* = 0.008) and better OS if chemotherapy was provided (HR 0.44, *p* = 0.006). Conversely, high baseline TSH x free T4 (>2.55) was associated with unfavorable TTTP (HR 2.03, *p* = 0.039) and overall survival (HR 3, *p* = 0.007) if sorafenib was administered. However, the association between this calculated ratio and thyroid status was not fully elucidated.

#### Effect on Head and Neck Cancer Survival

Head and neck cancer patients commonly undergo involved-field radiation therapy and are prone to iatrogenic hypothyroidism, which can therefore serve as a useful model to study the effect of thyroid dysfunction on cancer outcomes. Nelson et al. conducted a retrospective analysis of 155 patients with advanced head and neck squamous cell carcinoma who were treated with radiation therapy alone or in combination with other treatments (196). Patients who developed new onset hypothyroidism post-treatment had less cancer recurrence (*p* = 0.02), improved survival (*p* < 0.001), and longer recurrence-free survival (*p* < 0.001), compared with patients who did not. In another population based study of patients with head and neck cancer treated with radiotherapy (197), the 10 year incidence of hypothyroidism was 59% and these patients exhibited longer survival (HR 0.42, *p* < 0.001) as well as longer cause-specific survival (HR 0.36, *p* < 0.001). In a phase III trial comparing two cisplatin chemoradiotherapy protocols in 300 patients with locally advances head and neck cancer (222), 38.73% of patient developed hypothyroidism by 2 years of follow up (198). These patients had lower locoregional failure rate (LRFR) (hazard ratio 0.342, *p* = 0.043), and longer overall survival (hazard ratio 0.336, *p* = 0.001). Favorable impact on LRFR, PFS and OS were associated with hypothyroidism of longer duration and TSH levels up to 40 mIU/L.

### Association Between Thyroid Autoimmunity and Cancer

Small scale case control studies have demonstrated an increased prevalence of thyroid autoimmunity in breast cancer (223, 224), gastric cancer (225), pancreatic cancer (226), multiple myeloma (200), and myelodysplastic syndrome (203). In a 2012 meta-analysis, autoimmune thyroiditis, as well as overall thyroid antibody positivity, thyroglobulin antibody positivity and thyroid peroxidase antibody (TPOAb) positivity were associated with increased risk of breast cancer (OR 2.92, 2.02, 2.72, 2.64, respectively), with minimal to moderate heterogeneity (139). Conversely, in a population based case control study by Brandt et al. (128), including 676 breast cancer patients and 680 controls, women with high levels of TPOAb (above 9 kIU/L) were at a lower risk of being diagnosed with breast cancer (OR 0.75), specifically invasive type (OR 0.74).

Thyroid autoimmunity may beneficially affect cancer outcomes. Fiore et al. (227) examined the prognostic value of thyroid autoantibodies in 47 patients with locally metastatic breast cancer referred for mastectomy. Five-year mortality was lower in patients with thyroid autoantibody positivity (6.7 vs. 46.9%, *p* = 0.008). Farahati et al. assessed anti TPOAb in 314 patients with newly diagnosed breast cancer (228). Among 56 patients with TPOAb, no incidences of distant metastasis was documented, whereas in 17 (6.6%) of 258 cases without TPOAb, distant metastases were demonstrated (*p* = 0.04). In Brandt et al., high TPOAb levels were also associated with a lower risk of ductal cancer, large tumors (>20 mm), and ER and PR positive tumors (128). Interestingly, the same group recently identified several TPOAb related SNPs (s11675434, rs3094228, rs1033662, rs301806, and rs207140) which may also be associated with breast cancer risk (211). Another study by Franzke et al. included 329 patients with metastatic RCC treated with systemic IL-2 and IFNα2 (229). Antithyroid autoantibodies were detected in 18% of patients. Thyroid autoantibodies were correlated with increased survival (5-year survival 54 vs. 15%, *p* < 0.0001). Interestingly, HLA-Cw7 expression was more frequent with thyroid autoantibody positivity (69.2 vs. 47.7%, *p* = 0.009), and Cw7 expression was associated with prolonged overall survival, suggesting HLA-dependent thyroid autoimmunity associated with improved cancer outcomes. Thyroid autoantibodies may affect breast cancer behavior irrespective of its effect on the thyroid hormone axis. Thyroid cells and benign and malignant breast tissues share common antigens. The most important of these is the sodium-iodine symporter, which is highly expressed in breast cancer cells (230). Also, lactoperoxidase in breast cancer cells shares a homology with thyroperoxidase (231). T cells directed against thyroid autoantigens could attack breast cancer cells expressing similar antigens (227).

### Association of Cancer With Non-thyroidal Illness Syndrome (NTIS)

NTIS, or sick euthyroid syndrome, is characterized by alterations in circulating thyroid hormone levels in euthyroid patients with acute or chronic systemic illnesses. Changes include a decrease in T3 levels, increase in rT3 and inconstant alterations in circulating T4 levels (212). The association of NTIS with cancer was documented in various tumor types including breast cancer (232), gastrointestinal cancers (232), lung cancer (233–235), central nervous system tumors (236), multiple myeloma (237), chronic lymphocytic leukemia (238), and diffuse large cell lymphoma (239).

NTIS may be associated with adverse disease outcomes. In a 1978 study by Ratcliff et al. (233), 6 month mortality was higher among lung cancer patients with low T3 compared with matched lung cancer patients with normal T3 (49 vs. 27%). In a study of 80 patients with newly diagnosed non-small cell lung cancer (234), NTIS was more frequent among stage III (26%) and stage IV (62%) cases, and survival was shorter in patients with NTIS compared with patients without NTIS (mean survival 9.2 vs. 15.2 months, *p* = 0.00002). Similarly, in a cohort of both small cell and non-small cell cancer patients, NTIS was associated with disease stage and served as a poor prognostic factor (235). In a study of 230 patients with primary brain tumors (236), 27% had NTIS syndrome. Glioma patients with NTIS had greater 5-year mortality (HR = 2.197, *p* = 0.016) and shorter OS (249 vs. 352 days; *p* = 0.029). NTIS was also a predictor of poor post-operative outcomes in patients undergoing brain tumor surgery (240). In a study of patients with chronic lymphocytic leukemia (238), NTIS was associated with significantly shorter time to first treatment (2 vs. 11 months, *p* < 0.001) and cancer-specific survival (median survival 51 months vs. not reached) compared to patients with normal T3. In another study by the same group, of 188 patients with diffuse large B cell lymphoma, low T3 was associated with worse PFS (median survival 17 vs. 22 months) and overall survival (median survival 17 vs. 23 months) in the rituximab era (239). However, collectively these results may be a reflection of NTIS as a marker of aggressive disease, rather than a direct effect of this syndrome on cancer outcomes.

### The Association Between Thyroid Replacement Therapy and Cancer Incidence and Outcome

In an early study, 5,505 patients referred to a mammography department were interviewed regarding thyroid hormone use (150). Six hundred thirty-five patients used thyroid medications. In patients receiving thyroid supplements, breast cancer incidence was significantly higher than controls without thyroid disease or thyroid medication use (12.13 vs. 6.2%, *p* < 0.005). The difference was especially prominent in patients taking thyroid medication for more than 15 years (19 vs. 6.2%, *p* < 0.005) and nulliparous women taking thyroid medication for more than 5 years (20 vs. 9.2% in nulliparous controls, *p* < 0.025). These findings suggest a relationship between thyroid supplements and cancer associated with the duration of use. In a prospective cohort study, 2,738 post-menopausal women were screened for thyroid hormone parameters and prospectively followed for a period of 9 years (147). New breast cancer was related to previous use of thyroid medication at study inclusion (OR 3.2). However, a meta-analysis from 2017 including six studies evaluating the relationship between thyroid hormone supplementation and risk of breast cancer found no statistical correlation between the two (140). In a population based study, Cornelli et al. compared the prevalence of breast, colorectal, gastric and lung cancer in women during 2010 with the sales of levothyroxine (LT4) in the previous year in 18 Italian regions (161). Corrected for smoking and age, a significant correlation was demonstrated for lung cancer and levothyroxine sales (*R* = 0.485, *p* = 0.04). Sarosiek et al. performed a retrospective analysis in 504 pancreatic cancer patients who underwent a Whipple procedure or distal pancreatectomy and splenectomy during the course of 7 years (62). 14.1% of patients were hypothyroid. Hypothyroid patients taking exogenous thyroid hormone, in comparison to euthyroid patients, were more likely to have perineural invasion (OR 3.38, *p* = 0.012), have high T stage (T3-T4, OR 2.1, *p* = 0.045), nodal spread (OR 2.05, *p* = 0.018), and have poorer prognostic stage (2B-3, OR 1.89, *p* = 0.037). There was no difference in survival between both groups.

Similar to the apparent protective effect of endogenous thyroid hormones in colorectal cancer, exogenous thyroid hormone supplementation was associated with decreased risk of CRC. In a population based case control study conducted in northern Israel, 2,648 colorectal cancers were matched to 2,566 controls (187). Levothyroxine use for a minimum of 5 years, evaluated by structured interviews and prescription records validation, was associated with a significantly reduced risk of CRC (OR 0.59, *p* = 0.001). However, this study was limited by possible recall bias. Another large population based study compared 20,990 colorectal cancer patient with 82,054 matched control patients (184) and determined CRC risk in patients with thyroid dysfunction, with and without thyroid hormone replacement. Thyroid hormone supplementation use of more than 5 years was related with lower risk for CRC, with a stronger association documented for longer periods since initiation of treatment. The adjusted odds ratio for colorectal cancer associated with thyroid hormone replacement was 0.88 (*p* = 0.03) for treatment initiated 5–10 years before index date and 0.68 (*p* < 0.001) for treatment initiated more than 10 years before index date. In response to this study, Friedman et al. (188) performed a case-control analysis of colon (*n* = 12,207) and rectal/rectosigmoid cancers (*n* = 4,729), based on the Kaiser permanente cancer registry, and obtained LT4 prescription dispensing records from outpatient pharmacies. Each case patient was matched to up to 50 control subjects. Rectal cancer risk was more than 30% lower in men who used levothyroxine for more than 5 years, compared to non-users (OR 0.66, *p* = 0.03). Although statistically insignificant, colon cancer risk appeared to be somewhat reduced.

### The Effect of Induced Hypothyroidism and Hypothyroxinemia on Cancer Outcomes

Few interventional studies exist which examined the effect of chemically induced hypothyroidism and hypothyroxinemia on cancer outcomes. Those are based mainly on case reports and small patient series. A previous report described a patient with inoperable glioblastoma of the optic chiasm who failed standard treatment with radiation and temozolomide (169). The patient underwent induced hypothyroidism with PTU, followed by carboplatin chemotherapy. On two separate occasions, this patient responded clinically and radiographically to treatment with an extended remission period (2.5 years) and prolonged overall survival (4.5 years). In a recent report (183), a patient with triple negative breast cancer and lung metastasis who progressed under chemotherapy was treated with methimazole (45 mg per day) and increasing doses of liothyronine (L-T3). This treatment led to stabilization of the disease and CA-125 levels for several months. A second patient described in this study with metastatic pancreatic adenocarcinoma was treated with a similar protocol. In this case, treatment led to a temporary reduction in CA19-9 and a disappearance of a skin metastasis. In both patients, although L-T3 produced early resistance to treatment, a direct tumor growth inhibition effect was also observed. In a study published in 1976 (168), Yung et al. compared 32 patients with glioblastoma treated with surgery and radiation alone with 18 glioblastoma patients treated with surgery, radiation as well T3 to achieve a hyperthyroid state. Patients in the T3 treatment group had significantly longer median survival (60 vs. 30 weeks, *p* = 0.005). The authors speculated that this may be attributed to radiosensitizing property of triiodothyronine. Hercbergs et al. reported on 22 patients with recurrent glioma who were treated with PTU to induce hypothyroidism, concurrently with tamoxifen (170). Eleven patients became hypothyroid. Median survival was significantly longer in the hypothyroid group (10.1 vs. 3.1 months, *p* = 0.03). Lastly, another study by Hercbergs et al. included 23 patients with end stage cancers of the brain, ovary, lung, pancreas, salivary gland, and breast as wells mesothelioma and soft-tissue sarcoma (210). In euthyroid patients, hypothyroxinemia was reached by using methimazole, with the addition of L-T3 to avoid hypothyroidism side-effects parallel to suppressing endogenous TSH. The survival time of 83% (19 of 23) of patients exceeded the 20% expected 1-year survival for this group based on the SEER database (*p* < 0.01).

## The Effects of Thyroid Hormones on Angiogenesis and Anticancer Immune Responses

Generation of dense vasculature and evasion of immune reaction are among the key hallmarks of cancer (241). Recent studies provide evidence that TH affect both these crucial features of tumors, enabling cancer progression.

T4 acting on αvβ3 receptor stimulates formation of new vessels as shown in CAM assay and three-dimensional human microvascular endothelial sprouting model (97, 99, 100) which involves activation of MAPK (97). Inhibition of T4-αvβ3 signaling by tetrac or anti-integrin antibody blocked TH-induced formation of new vessels (97, 99). Importantly, tetrac and its nanoparticulate form also inhibited angiogenesis stimulated by cancer cells, as shown by CAM models implanted with renal cell cancer (59), medullary thyroid cancer (82), follicular thyroid carcinoma (83), and non-small cell lung cancer (40).

T4 may also facilitate cancer progression by interfering with anti-tumor immune responses. Treatment of breast and colon cancer cells with T4 stimulated the expression of PD-L1, one of the elements of PD-1/PD-L1 immune checkpoint that controls activation of T cells. Cancer cells often overexpress PD-L1 which interacts with PD-1 receptors on the surface of T cells, thus blocking its activation and attenuating immune response directed against tumors (242). In breast cancer cells, T4 activated PD-L1 expression via non-genomic mechanisms involving αvβ3 receptor. Remarkably, NDAT (nanotetrac) blocked T4-mediated activation of PD-L1, providing a possibility for restoring immune defense against cancer cells. One of the key drawbacks of cancer therapies directed against PD-1/PDL-1 checkpoint, aimed at activation of immune responses, is the risk of autoimmune disease in treated patients. Since αvβ3 is specifically overexpressed in cancer cells, and rarely functions on the surface of healthy cells, treatment of patients with NDAT would possibly not lead to autoimmune responses directed against non-cancer cells (16). Clearly, the results of these promising *in vitro* studies require further validation *in vivo* on a larger group of cancer types.

## Thyroid Hormones and Response to Therapy

The results of several *in vitro* studies suggest that thyroid hormones may influence responses to chemotherapy. Through promotion of cancer cell proliferation, mitochondrial activity, and cell cycle progression from G0-G1 to S, T3 enhances the sensitivity of breast cancer cells to various chemotherapies (22, 23). In pancreatic cancer cells T3 treatment potentiated cytotoxic activities of chemotherapeutics such as cisplatin or gemcitabine (63). Contrasting observations were made for cell lines derived from colonic tumors. In colon cancer cells T3 increases the expression of P-gp (MDR1), one of the key mediators of xenobiotic efflux (67, 109). In contrast to the above mentioned prevalent non-genomic TH effects stimulating cancer progression, T3 activates P-gp expression by TR binding to the direct repeat elements located upstream of the transcription start site of the P-gp gene (243). These results suggest that T3 may possibly interfere with drug treatments of colon cancer; however this hypothesis requires experimental verification. T4 and T3 also interfered with the activity of bortezomib, a key drug in treatment of MM patients (90).

## Conclusion

The thyroid hormones are increasingly acknowledged for their tumor-promoting effects. We aimed in this review to shed light on this association in order to clarify which types of cancer are thyroid-hormone sensitive and therefore are expected to favorably respond to manipulation of the thyroid hormone axis. This is highly relevant, specifically in the context of the discovery of the T4 receptor site upon the αvβ3 integrin, which is overexpressed in many tumor cells and vasculature and may serve as an attractive target for inhibition. However, currently few T4-αvβ3 inhibitors which demonstrated efficacy in several cancer types are under preclinical studies. Moreover, TH status is currently not yet considered as a risk factor or a prognostic factor in the clinical practice of cancer management, partly due to the conflicting data reported over the years. Future prospective studies to evaluate this link, with strict inclusion/exclusion criteria and predefined cut-off values, are therefore merited. Convincing results may promote the inclusion of thyroid status in the assessment of cancer patients and interventional studies may lead to novel treatment modalities that are desperately lacking in many aggressive cancers.

## Author Contributions

EK, AP-W, ME, and OA-F designed the review, collected the data, wrote, and approved the manuscript.

### Conflict of Interest Statement

The authors declare that the research was conducted in the absence of any commercial or financial relationships that could be construed as a potential conflict of interest.
