# Supplementary material for: Thyroid Hormones and Cancer: A Comprehensive Review of Preclinical and Clinical Studies
Source: Front Endocrinol (Lausanne). 2019 Feb 13;10:59. doi: 10.3389/fendo.2019.00059 (PMC6381772; doi:10.3389/fendo.2019.00059)
Supplement: Supplementary file 1 [file Data_Sheet_1.pdf]

Supplemental Table 1*: In vitro* studies on thyroid hormones and cancer

Cancer Type	Cell model	Treatment/ concentration	Thyroid hormone/inhibitor effect	Proposed mechanism	Reference
Breast	MCF7, T47D	T3/10^{-9}-10^{-7}M	T3 increased proliferation	ER dependent	(12)
	T47D	T3/10M	T3 increased proliferation	ER and T3R mediated	(13)
	MCF7	T3/10^{-11}-10^{-8}M	T3 increased proliferation	Thyroid receptor mediated	(10)
	MCF7 (ER+)	T3/10^{-10}-10^{-8}M	T3 increased proliferation	ER dependent	(14)
	MCF7	T4/10^{-7}M	T4 increased proliferation	Membrane receptor	(11)
	T47D	T3/10^{-9}M	T3 promoted cell migration	Integrin αvβ3	(17)
	MDA-MB-231	T4/10^{-7}M	T4 stimulated PDL1 expression	Integrin αvβ3	(18)
	MDA-MB-231	Nanotetrac/ 2X10^{-7}M	Nanotetrac sensitized to apoptosis and increased angiogenesis inhibitors	Integrin αvβ3	(19)
	MCF7	T3/10^{-6}M	T3 enhanced apoptosis	SMP30 downregulation	(206)
	MCF7	T3/7.7x10^{-6}-3.1x10^{-5}M T4/5X10^{-6}-2X10^{-5}M	T3 and T4 decreased proliferation	c-fos upregulation	(207)
	S30 (MDA-MB-231 ERα+)	T3/10^{-8}M	T3 inhibited proliferation	ER expression	(208)
	Hs 578T, MDA-MB-231, MDA-MB-468, T47D	T3/7.7x10^{-6}-3.1x10^{-5}M T4/5X10^{-6}-2X10^{-5}M	T3 and T4 inhibited proliferation	Undetermined	(209)
	MCF7 MDA-MB-231	T3/2-32 ng/ml	T3 enhanced chemosensitization to 5FU and taxol	Undetermined	(119)

	MDA-MB-231	T3/10^{-8}M	T3 enhanced doxorubicin-induced cell death	Sensitization of mitochondrial metabolism	(16)
Prostate	PC3, DU145, LNCaP	T3/7.7x10^{-6}-3.1x10^{-5}M T4/5X10^{-6}-2X10^{-5}M	T3 and T3 inhibited proliferation	c-fos upregulation	(207)
	LNCaP	T3/10^{-10}-10^{-6}M	T3 increased proliferation	Downregulation of BTG2	(20)
	LNCaP	T4/10^{-7}M	T4 induced invasion and VEGF		(21)
	LNCaP, PC3, DU145		T4 promoted migration and depressed detachment-induced apoptosis	Integrin αvβ3	(22)
Lung	H522	T4/10^{-7}M	T4 induced HIF-1α	Integrin αvβ3	(25)
	NCI-H522, NCI-H510A	T3/10^{-9}-10^{-6}M T4/10^{-8}-10^{-6}M	T4 and T3 increased proliferation	Integrin αvβ3	(23)
	H1299	T3/10^{-9}-10^{-6}M T4/10^{-8}-10^{-6}M	T4 and T3 increased proliferation	Integrin αvβ3	(24)
Ovary	OVCAR3	T3/7.7x10^{-6}-3.1x10^{-5}M T4/5X10^{-6}-2X10^{-5}M	T3 and T4 inhibited proliferation	Undetermined	(209)
	SKOV3, OVCAR3	T3, T4/10^{-5}-10^{-9}M	T3 and T4 Induced proliferation in SOKV3 and inhibited in OVCAR3	Akt pathway	(73)
	OVCAR3, A2780, SKOV3	T3/10^{-9}M T4/10^{-7}M	T3 and T4 increased proliferation	Integrin αvβ3	(26)
	OVCAR3, SKOV3	T4/10^{-7}M	T4 increased proliferation	Integrin αvβ3	(27)
	OVCAR3	T4/10^{-7}M	T4 induced HIF-1α	Integrin αvβ3	(25)
	OVCAR3, SKOV3, A2780	T3/10^{-9}M T4/10^{-7}M	T4 and T3 was involved in EMT	Integrin αvβ3	(28)
	OVCAR3, A2780	T3/10^{-9}M T4/10^{-7}M	T3 and T4 induced MAPK and PI3K mediated gene expression	Integrin αvβ3	(213)
	OVCAR3, A2780	Tetrac, Triac,T1AM/ 1-25x10^{-6}M	Tetrac,Ttriac and T1AM inhibited proliferation and induced apoptosis	Integrin αvβ3	(29)

Cervix	HeLa	T4/10^{-7}M	T4 induced MAPK activation	Membrane receptor	(32)
	HeLa	T4/2.3X10^{-11}M	T4 induced MAPK activation	Membrane receptor	(30)
	HeLa	T4/10^{-7}M	T4 induced MAPK activation	Membrane receptor	(31)
Glioblastoma	U87	T3,T4/ 10^{-10}-10^{-6}M	T3 and T4 increased proliferation	Integrin $\alpha v\beta 3$	(9)
	C6,F98, GL261	T3/10^{-8}-10^{-6}M T4/10^{-7}M	T4 and T3 increased proliferation	Integrin $\alpha v\beta 3$	(33)
	C6, GL261, U87MG	free T4/ 0.7 X10^{-10}M	T4 inhibited resveratrol induced apoptosis	Integrin $\alpha v\beta 3$	(34)
	U87MG	T3/10^{-9}M	T3 inhibited proliferation and promoted re-differentiation	Akt pathway	(35)
Astrocytoma	1321N1	T3/10^{-9}M	T3 increased proliferation and promoted re-differentiation	Akt pathway	(35)
Neuroblastoma	Neuroblastoma cells	T3/5x10^{-9}M	T3 inhibited ras-induced proliferation	Thyroid receptor mediated	(36)
Renal	Caki-2, Caki-1	T3/10^{-7}M	T3 increased proliferation	Thyroid receptor mediated	(42)
Gastric	MKN28	T3/2x10^{-9}M	T3 induced expression of VEGF and HIF1α	Akt pathway	(45)
Pancreas	hCM, hPANC1	T3, T4/ 10^{-5}-10^{-9}M	T3 and T4 increased proliferation in hCM and inhibited proliferation in hPANC1	Akt pathway	(73)
	MiaPaCa-2, AsPC-1	T3/10^{-9}-5x 10^{-6}M	T3 increased proliferation, migration, and invasion	Undetermined	(51)
	hPANC1, Capan1,	T3/ 10^{-5}-10^{-9}M	T3 inhibited proliferation and potentiated the cytotoxic effect of gemcitabine and cisplatin	Cyclin–CDK inhibition	(50)
Colon	HT-29, HCT116	T4/10^{-7}M	T4 stimulated PDL1 expression	Integrin $\alpha v\beta 3$	(18)
	HT29-D4	T3/1.5x10^{-9}M	T3 promotes limited cell growth and differentiation	Undetermined	(214)
	HT-29, COLO 205, HCT 116	T4/10^{-8}-10^{-6}M	T4 increased proliferation	Integrin $\alpha v\beta 3$	(46)

	LS180, Caco-2	T4/10^{-7}-10^{-4}M T3/2x10^{-9}-2X10^{-6}M	T4 and T3 up regulated MDR-1 protein	Undetermined	(48)
	CaCo-2	T3/3x10^{-9}M	T3 Inhibited proliferation and increased differentiation	E cadherin induction	(47)
	HT-29, HCT116	T4/10^{-7}M	T4 stimulated PDL1 expression	Integrin αvβ3	(18)
Hepatocellular	J7-TRα1	T3/10^{-8}M	T3 increased migration and invasion	Thyroid receptor mediated	(60)
	HepG2, Huh7, Mahlavu, J7	T3/10^{-8}M	T3 increased migration and invasion	Thyroid receptor mediated	(59)
	HepG2	T3/10^{-6}M	T3 induced ERK1/2 and Akt phosphorylation	Integrin αvβ3	(62)
	CSQT-2	T4/10^{-5}M	TH promoted HCC cells self-renewal	Thyroid receptor mediated	(61)
	HepG2-TR	T3/10^{-8}-10^{-7}M	T3 inhibited cell proliferation	Thyroid receptor mediated	(52)
	HepG2-TRα1, SK-Hep1-TRα1	T3/10^{-8}M	T3 reduced cell proliferation mediated by endoglin	Thyroid receptor mediated	(53)
	HepG2-TRα1	T3/10^{-8}M	T3 inhibited cell proliferation	Thyroid receptor mediated	(55)
	HepG2	T3/10nM	T3 upregulated DKK4 which suppressed invasion	Thyroid receptor mediated	(54)
Adrenocortical	SW13, H295R	T3, T4/ 10^{-9}-10^{-5}M	T3 and T4 Induced proliferation in SW13 and inhibited in H295R	Undetermined	(73)
Thyroid	BHP2-7, BHP18-21, FTC236	T3/10^{-10}-10^{-7}M T4/10^{-8}-10^{-7}M	T3 and T4 increased proliferation	Integrin αvβ3	(71)
Basal cell Carcinoma	G2N2C	T3/3x10^{-9}M	T3 reduced growth rate and induced apoptosis	Protein kinase A induction	(72)

Multiple Myeloma	RPMI 8226, CAG, primary BM cultures	T3/10^{-9}M<br>T4/10^{-7}M	T3 and T4 induced proliferation and viability	Integrin αvβ3	(64)
	RPMI 8226, U266, ARP-1, ARK, CAG	T4/10^{-7}-10^{-6}M	T3 and T4 induced proliferation and viability	Integrin αvβ3	(63)
	CAG, RPMI-8226, U266, primary BM cultures	T3/10^{-9}M<br>T4/10^{-7}M	T3 and T4 increased cell migration and invasion	Integrin αvβ3	(65)
	CAG , ARK,RPMI-8226, U266, ARP-1, primary BM cultures	Tetrac/<br>10^{-6}-5x10^{-5}M	Tetrac inhibited proliferation and induced apoptosis	Integrin αvβ3	(66)
Leukemia	LBC cells	T3/10^{-9}-10^{-4}M<br>T4/10^{-9}-10^{-4}M	No direct effect of thyroid hormones		(101)
Lymphoma	MJ , HuT78, OCI-Ly12, OCI-Ly13.2 , Mac2a, Karpas299 ,SUDHL1	T3/10^{-9}M<br>T4/10^{-7}M	T3 and T4 induced proliferation and VEGF expression	Integrin αvβ3	(69)

Angiogenesis	CAM model	T4/10^{-7}M	T4 induced angiogenesis	Integrin αvβ3	(199)
		T4/10^{-7}M T3/10^{-9}M	T3 and T4 induced angiogenesis	Membrane receptor	(216)
		T3,T4/ 10^{-7}-10^{-9}M	T4 and T3 induced angiogenesis	Integrin αvβ3	(201)
		T4/10^{-7}M	T4 induced angiogenesis	Integrin αvβ3	(200)
	CAM (lung cancer)	Tetrac 1µgr/CAM	Tetrac inhibited tumor related angiogenesis	Integrin αvβ3	(24)
	CAM (renal cell cancer)	Tetrac 1µgr/CAM	Tetrac inhibited tumor related angiogenesis	Integrin αvβ3	(202)
	CAM (Medullar y thyroid cancer)	Tetrac 1µgr/CAM	Tetrac inhibited tumor related angiogenesis	Integrin αvβ3	(92)
	CAM (follicular cancer)	Tetrac 1µgr/CAM	Tetrac inhibited tumor related angiogenesis	Integrin αvβ3	(203)

Supplemental Table 2*: In vivo* studies on thyroid hormones and cancer

Cancer type	Animal model	Cancer model	Thyroid hormone/inhibitor effect	References
Breast	C3H/HeN mice	Mammary tumor virus	T3 increased tumor incidence	(210)
	Sprague-dawley rats	MNU induced breast cancer	Hyperthyroidism increased tumor incidence. Hypothyroidism decreased tumor incidence	(211)
	C3H/HeN mice	Mammary tumor virus	Hypothyroidism decreased tumor incidence	(77)
	Sprague-dawley rats	DMBA induced breast cancer	Hypothyroidism decreased tumor incidence	(80)
	Sprague-dawley rats	DMBA induced breast cancer	Hypothyroidism decreased tumor incidence, increased latency and slowed tumor growth	(82)
	C3H/he mice	C3HBA cells	hypothyroidism slowed tumor growth and increased survival	(83)
	Sprague dawley rats	MNU induced breast cancer	Hypothyroidism decreased tumor incidence and tumor volume	(81)
	CH3/He mice	C3HBA cells	Hypothyroidism led to tumor remission	(84)
	C3H-A mice	De novo	Hyperthyroidism increased tumor incidence and aggressiveness. Hypothyroidism decreased tumor incidence	(212)
	Nu/Nu mice	MDA-MB-468	Hypothyroidism retarded tumor growth and enhanced invasiveness and metastases	(85)
Prostate	Nu/Nu mice	PC3 cells	Hypothyroidism reduced growth rate	(87)
	Ncr-nude mice<br>mnNCr-nude mice	PC3 cells	Hypothyroidism reduced growth rate	(88)

	BALB/c mice	LNCaP, DU145 cells	T3 reduced tumor growth	(21)
Lung	C57BL/6N mice	3LL cells	T4 increased tumor growth and metastases	(89)
	CB4-C57 Mice	3LL cells	T4 increased tumor growth and angiogenesis	(90)
	NCr nude mice	NCI-H1299 cells	Tetrac suppressed tumor growth	(24)
	mnNCr-nude mice	201T cells	Hypothyroidism slowed tumor growth	(88)
Glioblastoma	Nude NCr mice	U87MG-luc cells	Nanotetrac reduced tumor size and decreased vascularity	(93)
Renal	NCr nude mice	CR1932-786-0 cells	Tetrac reduced tumor size	(202)
Gastric	Wistar rats	MNNG induced breast cancer	Hyperthyroidism increased cancer incidence	(94)
Pancreas	NCr mice	PANC-1, MPanc96 cells	Tetrac inhibited tumor growth and angiogenesis	(91)
Colon	Wistar rats	Azoxymethane (AOM) induced colon cancer	T4 increased cancer incidence	(95)
Hepatocellular	Buffalo rats	Morris Hepatoma 44 cells	Hypothyroidism reduced tumor growth, number and size of metastases and prolonged survival.	(96)
	Fisher rats	DENA/2-AAF induced hepatocarcinoma	Hyperthyroidism associated with preneoplastic nodule regression	(56)
	Scid mice	J7-TR cells	T3 increased invasion and lung metastases	(60)
	Nu/Nu mice	SK-hep1	Hypothyroidism retarded tumor growth and enhanced invasiveness and metastases	(85)
Thyroid	NCr nude mice	h-MTC cells	Tetrac inhibited tumor growth	(92)
	$Thrb^{PV/PV}$	De novo	Hypothyroidism inhibited tumor growth	(108)
	NCr nude mice	FTC-236	Tetrac inhibited tumor growth	(203)
Melanoma	C57BL/6 mice	B16F10 cells	Hypothyroidism increased tumor latency and survival	(103)

Basal cell carcinoma	K5-Gli2	De novo tumors	T3 reduced tumor growth	(72)
Ehrlich tumor carcinoma	Swiss mice	Ehlich tumor cells	Hyperthyroidism increased tumor size	(78)
	Swiss strain mice	Ehrlich tumor cells	Hypothyroidism resulted in lower ascitic volume	(215)
Sarcoma	A/Jax mice C57BL/6J mice	S-1, t241 cells	Thyroxine tumor size and metastases.	(106)
Lymphoma	C57BL/6J mice	EL4 cells	Hyperthyroidism increased tumor growth	(70)
	C57BL/6J mice	EL4 cells	Hypothyroidism increased tumor growth and reduced survival	(102)

Supplemental table 3: Clinical studies on thyroid hormones and cancer

Cancer type	Research design	Number of cases	Thyroid function/ treatment	Clinical outcome	Reference
Breast	Case control	682 pts<br>731 controls	Full thyroid spectrum	Higher total T4 and lower total T3 associated with increased risk	(118)
	Population based	75,056	hyperthyroidism	Elevated risk and mortality after 60 years	(142)
	Meta analysis	24,808<br>(13 studies)	Hypothyroidism/ Hyperthyroidism	Thyroid dysfunction may not be related to cancer risk	(122)
	Population based	61,873 hypothyroid<br>80,373 hyperthyroid	Hypothyroidism/ Hyperthyroidism	Hyperthyroidism associated with increased and hypothyroidism associated with decreased risk	(115)
	Series/cohort	437	Full thyroid spectrum	Higher TSH associated with decreased risk of triple positive breast cancer	(116)
	Case control	676 pts<br>680 controls	Hyperthyroidism	High free T4 associated with higher risk	(113)
	Case control	120	Full thyroid spectrum	Higher total T4 and total T3 in cancer patients	(230)
	Clinical trial	28	hypothyroidism	Longer progression free survival	(150)
	Population based	2,185	Hyperthyroidism	Higher T3 associated with higher risk of cancer, large tumors and lymph node metastases	(114)
	Case control	2,663 pts<br>2,344 controls	Thyroid disorders	Thyroid disease unrelated to cancer risk	(223)

	Case control	4,575 pts<br>4,682 controls	Thyroid disorders	Thyroid disorders unrelated to cancer risk	(220)
	Case control	1,362 patients<br>1,250 controls	Hypothyroidism	Reduced cancer risk	(221)
	case control	369 pts<br>316 controls	range	Lower total T3 and higher total T4 in cancer patients	(119)
	Case control	676 pts<br>680 controls	Hyperthyroidis m	Increased cancer risk	(112)
	Population based	2,186	Hyperthyroidism	T3 associated with breast cancer death	(149)
	Meta analysis	18,428 cases<br>22,378 controls<br>(26 studies)	Hypothyroidism/ Hyperthyroidism	No significant association with breast cancer risk	(121)
	Meta-analysis	206,918<br>(12 studies)	Hypothyroidism	No association with breast cancer risk	(224)
	Population based	2,696	Hyperthyroidism	T3 associated with cancer risk	(219)
	Case control	65 pts<br>38 controls	Full thyroid spectrum	Free T3 and free T4 higher in cancer patients	(217)
	Series/cohort	1,665	Hypothyroidism	No increased risk in hypothyroid women using thyroid supplements	(226)
	Series/cohort	5,505	Undetermined	Higher incidence of cancer in patients receiving thyroid supplements	(188)
	Series/cohort	2,775	Hypothyroidism	Cancer related to earlier hypothyroidism, low free T4 and use of thyroid medication	(189)

	Case control	21 cancer and hyperthyroidism<br>100 cancer without thyroid dis	hyperthyroidism	Higher mortality in hyperthyroid patients	(148)
	Population based cohort	89,793 LT4 users<br>89,793 controls	LT4 treatment	Lower mortality followin BC diagnosis in LT4 users.	(225)
	Case control	1,136 pts<br>1,088 controls	hypothyroidism	Lower risk of BC, older age at diagnosis, more localized disease, less lymph node involvement	(137)
	Population based	10,318	Hyperthyroidism	Higher free T4 associated with higher risk	(111)
	Case control	26 pts<br>22 controls	Subclinical hyperthyroidism	Higher subclinical hyperthyroidism in postmenopausal cancer patients	(218)
Prostate	Series/cohort	68	Hyperthyroidism	T3 associated with risk of recurrence	(227)
	Case control	402 pts<br>800 controls	hypothyroidism	Decreased risk	(124)
	Case control	161 pts<br>27 controls	Full thyroid spectrum	Higher T3 in patients	(228)
	Series/cohort	3,649	Hyperthyroidism / Hypothyroidism	Higher TSH associated with lower risk. Higher free T4 associated with increased risk	(123)
	Population based	29,691	Hyperthyroidism	Increased cancer risk	(109)
Lung	Series/cohort	51	Hypothyroidism	Longer surival	(152)

	Case control	85 pts<br>85 controls	Hypothyroidism	Longer survial and later diagnosis	(151)
	Population based	13.93 million LT4 boxes	LT4 treatment	LT4 use associated with cancer	(190)
	Series/cohort	7338 women	Hyperthyroidism	Increased mortality	(231)
	Population based	10,318	Hyperthyroidism	Higher free T4 associated with higher risk	(111)
	Population based	29,691	Hyperthyroidism	Increased risk	(109)
	Case control	9 pts<br>11 controls	Full thyroid spectrum	TSH lower and free T4 higher in cancer pts	(229)
Ovarian	cohort	238,130	Hypothyroidism	Inverse association with cancer (nonsignificant)	(126)
	Case control	120	Full thyroid spectrum	Higher T4 in cancer patients	(230)
	Case control	767 pts<br>1,367 controls	hyperthyroidism	Increased risk	(125)
	Population based case-control	160 hyperthyroidism<br>624 hypothyroidism<br>5038 control	Hyper/hypothyroidism	Hyperthyroidism associted with increased mortality. Modest association with hypothyroidism.	(153)
Uterine	Series/cohort	238,130	Hypothyroidism /hyperthyroidism	Hyperthyroidism positively and hypothyroidism inverselty associated with cancer (nonsignificant)	(126)
	observational	199 patients with EC	Hypothyroidism	Decreased survival	(232)

	Population based	99,812	Thyroid disease	Uterine cancers related to previous thyroid disease	(127)
Central nervous system	Case control	18 cases<br>32 controls	T3 treatment	Longer survival in T3 treated glioblastoma patients	(195)
	Interventional case control	11 cases<br>11 controls	PTU induced hypothyroidism	Longer survival in hypothyroid group	(196)
	Case report	1	Induced hypothyroidism	Prolonged survival	(193)
Renal	Meta analysis	147<br>(4 studies)	Hypothyroidism	Improved survival in hypothyroid patients with sunitinib therapy	(163)
	Series/cohort	65	Hypothyroidism	Improved survival	(157)
	Prospective observational	111<br>(sunitinib tx.)	hypothyroidism	No difference in PFS after 6m	(162)
	Series/cohort	31 (sunitinib tx.)	Hypothyroidism	Longer survival (nonsignificant)	(234)
	Series/cohort	41 (sunitinib tx.)	Hypothyroidism	Prolonged PFS in hypothyroid patients receiving thyroid substitution	(158)
	Series/cohort	83 (TKI tx.)	Hypothyroidism	Prolonged PFS	(160)
	Series/cohort	16	hypothyroidism	Favourable response to treatment	(154)
	Series/cohort	87 (TKI'S)	Subclinical hypothyroidism	Increased remission and overall survival	(159)
	Series/cohort	22 (sunitinib Tx)	hypothyroidism	Longer PFS	(161)

	Series/cohort	17 sunitinib tx	Hypothyroidism (>50% reduction in thyroid gland)	Longer PFS	(235)
	Population based	57326	Hypothyroidism	Increased risk in women	(233)
Esophageal	Case control	102 pts<br>160 controls	hyperthyroidism	Higher incidene of hyperthyroidism in cancer group	(236)
Pancreas	Series/cohort	504	LT4 treatment	Higher perineural invasion, T stage, nodal spread and poorer prognostic stage	(51)
	Case control	532 pts<br>1,701 controls	hyperthyroidism	Increased risk	(128)
	Case report interventional	1	Induced hypothyroxinemi a	Tumor regression	(194)
	Series/cohort	7338	Hyperthyroidism	Increased mortality	(231)
Colorectal	Case control	2,566 pts<br>2,566 controls	undetermined	Eltroxin use associted with reduced risk of CRC	(191)
	Case control	273 pts<br>819 controls	Subclinical hypothyroidism	Higher subclinical hypothyroisim in crc group	(139)
	Prospective cohort	3,836	Full thyroid spectrum	Higher TSH associated with increased risk	(130)
	Cohort	68	Free T3/free T4 ratio	Higher baseline ratio associated with increased survival	(164)
	Case control	4729 pts<br>235925 controls	LT4 treatment	Reduction of rectal cancer risk associated with long term use of eltroxin	(192)
	Case control	20,990 pts<br>82,054 controls	Hyperthyroidism /hypothyroidism/ LT4 treatment	Hyperthyroidism and untreated hypothyroidism associated with	(129)

				increased risk and Long term thyroid hormone replacement associated with decreased risk of crc.	
Hepatocellular	Case control	420 pts<br>1104 controls	hypothyroidism	Increased risk in women	(131)
	Series/cohort	123	TSH x free t4	Higher value associated with favourable survival if chemotherapy provided and unfavourable if sorafenib administered.	(165)
	Case control	54 cases<br>106 controls	hypothyroidism	Hypothyroidism more prevalent in HCC patients with unknown etiology	(132)
	Series/cohort	667	Hypothyroidism/ Hyperthyroidism	High TSH associated with larger tumor and low TSH associated with smaller tumors. Elevated free T4 assocaited with lower overall surival.	(140)
	Population based	212,456	Full thyroid spectrum	Free T3 and free T4 inversley associated with cancer mortality	(146)
Thyroid	Population based	57,326	Hyperthyroidism	Increased risk	(233)
	Case control	17,033 hyperthyroidi sm<br>34,066 controls	hyperthyroidism	Increased risk	(110)
Head and neck	Series/cohort	155	Hypothyroidism	Increased survival	(166)
	Population based	5,916	Hypothyroidism	Increased survival	(167)

	Prospective randomized	300	hypothyroidism	Increased survival	(169)
Melanoma	Series/cohort	91	Hypothyroidism	No difference in survival	(237)
Multiple myeloma	Case control	73 pts<br>73 controls	Full thyroid spectrum	Free T3 higher and TSH lower in patients	(134)
Acute leukemia	Case control	25 pts<br>25 controls	Full thyroid spectrum	Free T3, free T4, total T3, total T4 higher and TSH lower in cancer patients	(136)
	Series/cohort	870	Hyperthyroidism /hypothyroidism	Improved outcome in Graves dis and Hashimoto	(238)
Myelodysplastic syndrome	Case control	101 pts<br>101 control	Full thyroid spectrum	Free T3 and free T4 higher and TSH lower in patients	(135)
Hematopoietic	Population based	57,326	Hypothyroidism	Increased risk in women	(233)
General	case control	2793 hyperthyroid<br>2793 controls	Hypothyroidism	Hypothyroidism decreased cancer mortality followin RAI tx.	(241)
	Series/cohort	7338	Hyperthyroidism	Increased cancer mortality	(231)
	Population based	3651	Hyperthyroidism	No association with cancer mortality	(239)
	Case control	3052 hyperthyroidism<br>12208 controls	Hyperthyroidism	TNG associated with increased cancer mortality	(141)
	Case series	23 end stage tumor patients	Induced hypothyroxinemia	Prolonged survival	(197)

	Population based	115,746	Subclinical hypothyroidism	Higher cancer mortality (specifically bone, skin and breast)	(145)
	Case control	2004 pts<br>10111 controls	Subclinical hyperthyroidism	No increased risk of cancer mortality	(240)
	Population based	212,456	Full thyroid spectrum	Free T3 inversely associated with cancer mortality	(146)
	Population based	57,326	Hypothyroidism	Increased risk of cancer 10 years after diagnosis	(233)
	Series/cohort	24	hypothyroidism	High response rate to radiation therapy	(243)
	Series/cohort	538 (TKI tx)	Hypothyroidism/ LT4 treatment	Hypothyroidism improved survival<br>In 5<TSH<10, hormone replacement improved survival	(242)
	Case control	457 cases<br>373 controls	Hyperthyroidism /Hypothyroidism	Hypothyroidism and hyperthyroidism more common in cancer patients	(179)
	Population based	29,691	Hyperthyroidism	Increased cancer risk	(109)
	Population based	10,318	Hyperthyroidism	Free T4 associated with increased cancer risk	(111)
	Case control	17,033 hyperthyroidism<br>34,066 controls	Hyperthyroidism	Increased cancer risk	(110)
	Case control	9,520 pts<br>9,520 controls	Hypothyroidism	Lower cancer mortalithy in hypothyroid patients	(222)
	Series/cohort	17	Hypothyroidism	Higher rate of tumor regression	(138)
